# Interleukin-1 mediates innate immune signaling and antiviral defense in islets

**DOI:** 10.26508/lsa.202603795

**Published:** 2026-08-25

**Authors:** Jacob T Bartosiak, Polly A Hansen, Khanmi Kasomva, Sridhar Rao, John A Corbett

**Affiliations:** 1 Department of Biochemistry, Medical College of Wisconsin , Milwaukee, WI, USA; 2 Division of Hematology and Oncology, Department of Medicine, Medical College of Wisconsin , Milwaukee, WI, USA; 3 Hematopoiesis and Immunology Program, Versiti Blood Research Institute, Milwaukee, WI, USA; 4 Department of Cell Biology, Neurobiology and Anatomy, Medical College of Wisconsin , Milwaukee, WI, USA; 5 Division of Hematology/Oncology/Transplantation, Department of Pediatrics, Medical College of Wisconsin , Milwaukee, WI, USA

## Abstract

Based on in vitro studies, cytokines are believed to contribute to the pathogenesis of type 1 diabetes by directly causing β-cell damage. In this study, the effects of endogenously produced cytokines, following lipopolysaccharide (LPS) administration to mice, on gene expression in islet cells were evaluated by single-cell RNA sequencing (scRNA-seq). In response to LPS, there is an increase in antiviral and antibacterial genes and repression of genes associated with identity in all endocrine cell populations. While islet-resident non-lymphoid hematopoietic cells were not classically activated, antiviral and antibacterial gene expression was increased. Antipathogen gene expression was also increased in the islet endothelium, while genes associated with transcription, cell migration, and adhesion were decreased. IL-1 was identified as the primary mediator of changes in β-cell gene expression, and we show that IL-1 limits picornavirus replication in islets. Overall, these findings support a role for IL-1-stimulated antipathogen gene expression in β-cells as a physiologically relevant immune-endocrine signaling axis that functions to enhance fitness and reduce susceptibility of these essential cells to virus-mediated damage.

## Abstract (precis)

In vivo innate immune activation results in IL-1-dependent expression of antipathogen defense response genes and attenuates picornavirus replication in islets.

## Graphical Abstract

**Figure:**
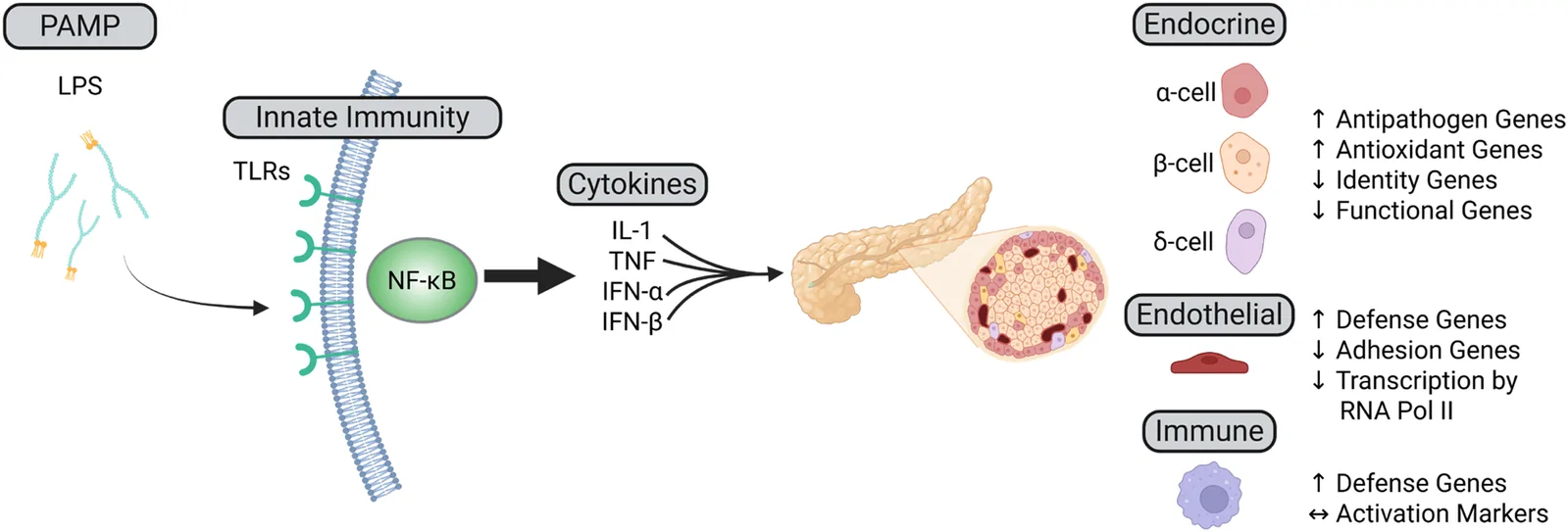

## Introduction

The mechanisms responsible for the initiation of autoimmunity directed against β-cells have yet to be fully identified; however, cytokine-mediated β-cell damage is believed to contribute to this process (1). In support of this hypothesis, in vitro exposure to interleukin-1 (IL-1), alone or in combination with other cytokines, such as tumor necrosis factor α (TNF-α) or interferon-γ (IFN-γ), results in a time- and concentration-dependent inhibition of insulin secretion that is followed by the degeneration of islets (2, 3). These effects are dependent on expression of the inducible form of nitric oxide synthase (iNOS) and the production of micromolar concentrations of nitric oxide (NO) (4, 5, 6). It is through the inhibition of complex IV of the electron transport chain and aconitase of the Krebs cycle, events that decrease β-cell ATP levels, that NO inhibits insulin secretion (7, 8, 9, 10, 11). Although not often discussed, the actions of cytokines and NO are reversible, either by removing the cytokine by washing and continued culture, or by inhibiting NO production (in the presence of the cytokines) (12, 13, 14); however, prolonged culture with cytokines beyond 36 h duration results in an irreversible inhibition of function and commitment to islet degeneration (12, 13). Cytokine and NO exposure have also been reported to induce ER stress and activate the unfolded protein response, induce DNA damage, and stimulate apoptosis (15).

The transcriptional responses of endocrine cells to cytokines have been extensively characterized by single cell RNA-sequencing (scRNA-seq) using mouse, rat, and human islets (16, 17, 18, 19, 20). Most of these studies have been performed following cytokine exposure that correlates temporally with islet damage and destruction (24, 48 h incubations) and unsurprisingly have identified changes in gene expression associated with the induction of cellular stress, islet inflammation, and apoptosis (16, 17). Additional studies have reported up-regulation of HLA class I and modified alternative splicing that have been proposed to contribute to neoantigen generation and presentation during T1D onset (19). We have taken a different approach and focused on early transcriptional responses of mouse and human islets to in vitro cytokine exposure of 6–18 h (21, 22, 23). Rather than increased pro-apoptotic or ER-stress-associated gene expression, we observed enrichment of transcriptional programs including antiviral, antibacterial, and antioxidant pathways (21, 22, 23). These findings suggest a physiological, antipathogen role for cytokine signaling in islets; consistent with this view, it has been shown that NO inhibits viral replication and apoptosis and promotes the activation of DNA repair pathways in β-cells (24, 25, 26, 27, 28, 29).

While much information has been gained from in vitro exposure of islets to cytokines, these studies fall short of mimicking the transient changes in cytokine levels that occur following infection or injury. In response to pathogen-associated molecular patterns (PAMPs) or damage-associated molecular patterns (DAMPs) during times of infection or tissue damage, serum cytokine concentrations are transiently elevated. Islets are highly vascularized, receiving up to 20% of the pancreatic blood flow (30), and are exposed to circulating soluble mediators in addition to blood glucose. Recently, we developed an experimental model system to study islet responses to endogenously produced cytokines. Using a low, non-septic dose of lipopolysaccharide (LPS) to stimulate cytokine production in mice (31), we observed a transient shift in transcriptional programming in islets consistent with early cytokine responses observed in vitro, including increased antipathogen and decreased identity gene expression (21). The transcriptional responses peaked at ∼6 h and returned to control levels 24-h post LPS administration (21). While serum levels of multiple cytokines are elevated following LPS administration, the expression levels of several antiviral genes were decreased in mice with a β-cell-specific deletion of IL-1R (βIl1r1KO mice) (21).

In this study, the transcriptional responses of islet endocrine and non-endocrine cells to endogenously produced cytokines in vivo were evaluated. We show that defense response genes are the most highly enriched categories of genes that increase in expression in β-cells and other islet endocrine cells following PAMP administration to mice. We did not identify changes in the expression of genes associated with stress responses, apoptosis, or cellular damage. βIl1r1KO mice were used to identify a primary role for IL-1 signaling in regulating the expression of antipathogen genes in β-cells, including the expression of antiviral genes known to be controlled by IFNs in most cell types. Consistent with the induction of antiviral activities, we show that IL-1 exposure attenuates the replication of a diabetogenic picornavirus, encephalomyocarditis virus (EMCV), in islets as effectively as IFN-γ. These findings support the hypothesis that soluble mediators, produced by the innate immune system in response to PAMPs, serve as alarm signals of potential danger and function to initiate defense responses and prepare islets for potential insult.

## Results

### Single-cell RNA sequencing of islet cells isolated 6 h after administration of LPS to C57BL/6J mice

**Figure 1.**
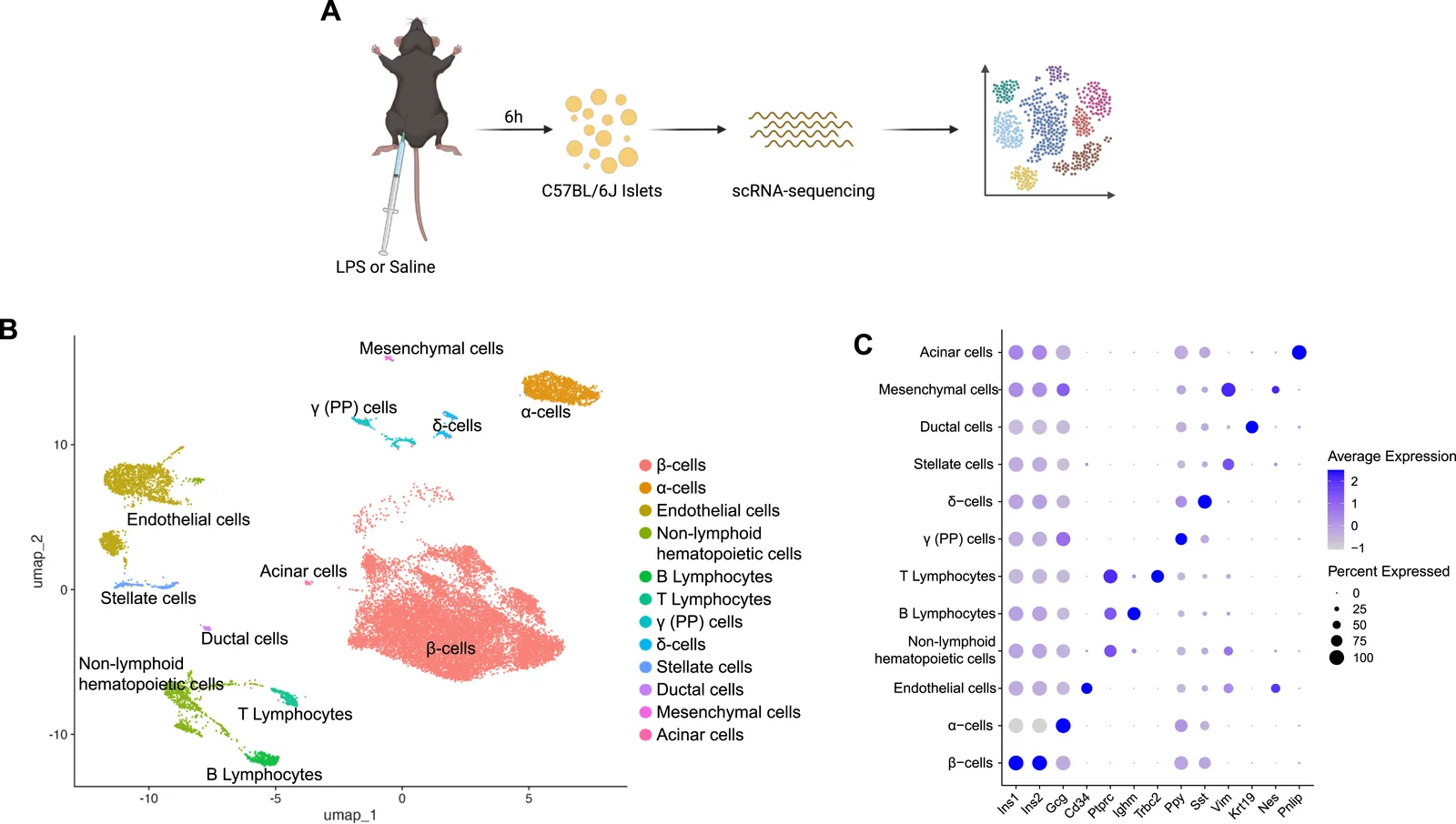
Single-cell RNA sequencing of islet cells following LPS administration to C57BL/6J mice. **(A)** Experimental design schematic. **(B)** Uniform Manifold Approximation and Projection (UMAP) plot color-coded by cell identity based on characteristic gene expression. **(C)** Dot plot indicating expression of select marker genes in each cell population.

**Figure S1.**
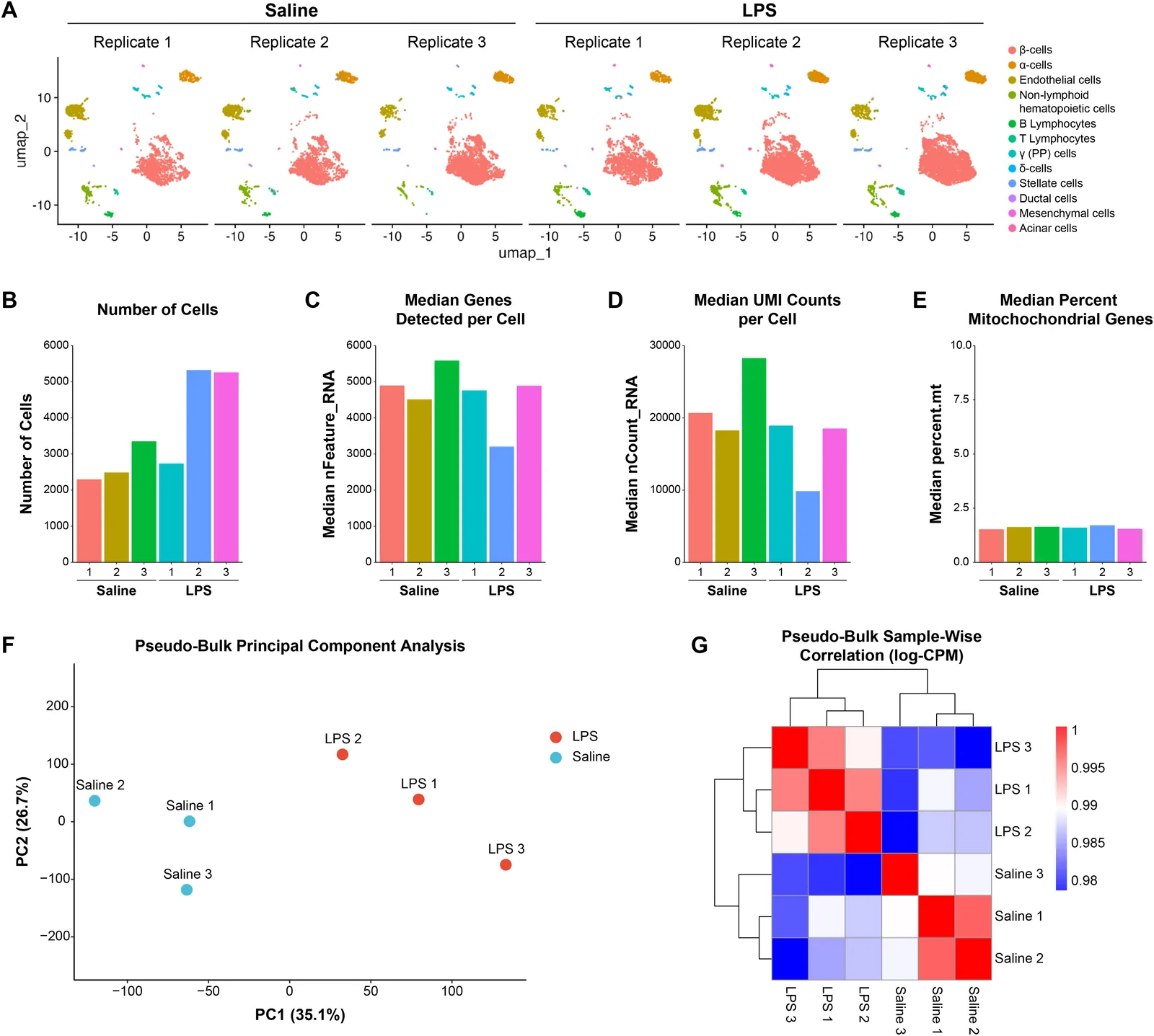
C57BL/6J scRNA-seq replicate consistency. **(A)** Uniform Manifold Approximation and Projection (UMAP) plots split by each biological replicate and color-coded by cell identity. **(B)** The number of cells, **(C)** median number of detected genes (nFeature_RNA), **(D)** median unique molecular identifier (UMI) counts (nCount_RNA), and **(E)** median percent mitochondrial genes (percent.mt) per sample. **(F)** Pseudo-bulk expression profiles were generated by summing raw UMI counts, normalizing to counts-per-million (CPM), and applying log1p transform. Principle component analysis of pseudo-bulk samples is shown. **(G)** Sample-wise Pearson correlation coefficients with dendrogram.

To understand the physiological responses of individual islet cell populations to endogenous cytokines produced following innate immune activation by PAMP treatment, single cell RNA-sequencing was performed. Islets were isolated from C57BL/6J mice 6 h after intraperitoneal administration of LPS (0.33 mg/kg) or endotoxin-free physiological saline (Fig 1A). The time and dose of LPS used for these studies were based on endogenous cytokine production, cytokine-dependent gene expression in islets, and the induction of mild transient illness behavior (31, 32). Cells from six samples (three biological replicates per condition) were visualized with a Uniform Manifold Approximation and Projection (UMAP) plot (Fig 1B), and cell identity was assigned to each cluster based on characteristic gene expression (Fig 1C). Cell number and percentage of total viable cells are given: β-cells (*Ins1*, *Ins2* – 14,290 cells, 71%), endothelial cells (*Cd34* – 1,995 cells, 9.9%), α-cells (*Gcg* – 1,405 cells, 6.9%), non-lymphoid hematopoietic cells (*Ptprc* – 908 cells, 4.5%), B-lymphocytes (*Ptprc*, *Ighd* – 355 cells, 1.7%), T-lymphocytes (*Ptprc*, *Trbc2* – 287 cells, 1.4%), PP (γ) cells (*Ppy* – 301 cells, 1.5%), stellate cells (*Vim* – 293 cells, 1.4%), δ-cells (*Sst* – 191 cells, <1%), ductal cells (*Krt19* – 91 cells, <1%), mesenchymal cells (*Vim*, *Nes* – 69 cells, <1%), acinar cells (*Pnlip* – 49 cells, <1%). Individual samples were evaluated, as shown in Fig S1A–G and determined to have a high degree of consistency across biological replicates.

### Defense response genes are enriched in β-cells from LPS-treated mice

**Figure 2.**
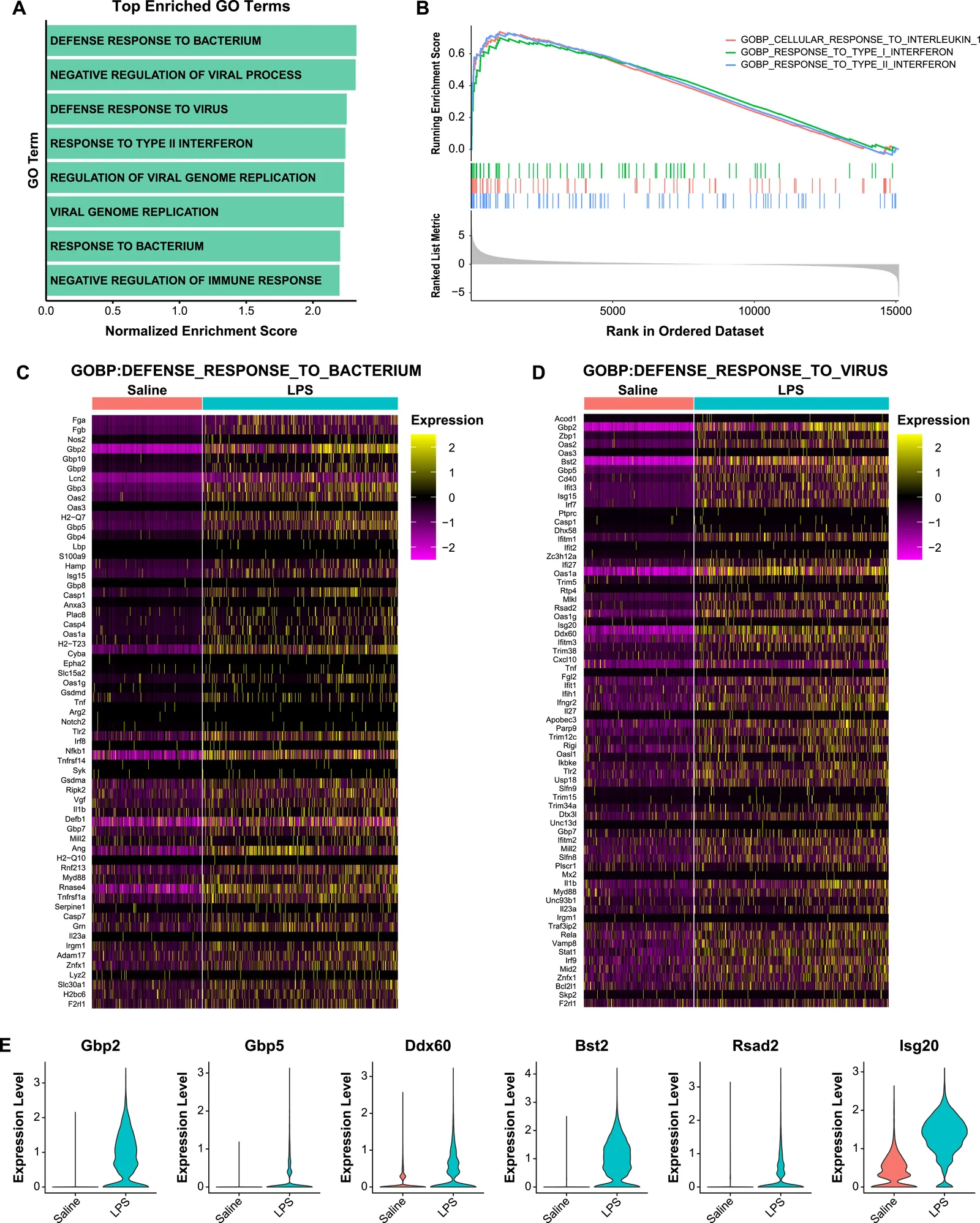
β-cell genes with enhanced expression following LPS administration to mice. **(A)** Most highly-enriched GO terms in β-cells isolated 6 h after LPS administration to mice was determined by gene set enrichment analysis (GSEA) using the *MSigDB C5: Ontology Gene Set* curated by the BROAD Institute. **(B)** Enrichment plot for the GO terms “Response to Type II Interferon” (NES = 2.24, *P*-adj = 8.77e−09), “Cellular Response to Interleukin-1” (NES = 2.15, *P*-adj = 1.76e−07), and “Response to Type I Interferon” (NES = 2.04, *P*-adj = 7.53e−06). **(C)** Heat map depicting β-cell expression of genes with the GO term “Defense Response to Bacterium” or **(D)** “Defense Response to Virus.” **(E)** Violin plots showing the expression level of select genes in β-cells isolated from mice after LPS or saline administration.

To determine the β-cell response to innate immune activation and endogenous cytokine production, the β-cell cluster was computationally isolated and differentially expressed genes, comparing the LPS- and saline-treated mice, were identified. Genes were rank ordered by increasing adjusted *P*-value (*P*-adj) and gene set enrichment analysis (GSEA) was performed using the *MSigDB C5: Ontology Gene Set* curated by the BROAD Institute (33, 34). Seven of the top eight most highly enriched gene categories, by normalized enrichment score (NES), are involved in defense responses to pathogens (Fig 2A). This includes the two most highly enriched gene categories of “Defense Response to Bacterium” and “Negative Regulation of Viral Process.” These findings suggest that the β-cell transcriptional response to endogenous cytokine signaling is an increase in the expression of genes with known roles in defense against pathogen-mediated damage. These findings are consistent with our previous studies that report up-regulation of antiviral responses in β-cells following a 6 h in vitro culture of islets with IL-1 and/or IFN-γ (21). As shown in Fig 2B, the enrichment of cytokine response categories including “Response to Type II Interferon” (NES = 2.24, *P*-adj = 8.77e−09), “Cellular Response to Interleukin-1” (NES = 2.15, *P*-adj = 1.76e−07), and “Response to Type I Interferon” (NES = 2.04, *P*-adj = 7.53e−06) suggests that β-cells are likely responding to signaling from multiple different cytokines to coordinate a cellular defense response. This defense response includes up-regulation of genes known to contribute to defense response to bacterium (Fig 2C) and defense response to virus (Fig 2D). These genes have been shown to interfere with bacterial or viral infection and contribute to host defense against diverse pathogens through a variety of mechanisms. For example, *Bst2* (Fig 2E), one of the most highly up-regulated genes in β-cells from LPS-treated mice, encodes for the protein tetherin, which functions to restrict the spread of cell-free virions and protects against enveloped viruses containing negative-sense RNA, positive-sense RNA, or dsDNA by anchoring virions to the cell membrane of infected cells (35). Guanylate binding proteins 2 (GBP2) and 5 (GBP5) also exhibit broad antiviral activity, including inhibition of encephalomyocarditis virus (EMCV) replication by GBP2 (36, 37). EMCV is known to cause β-cell lysis and to induce diabetes in genetically susceptible strains of mice (38) suggesting these antiviral effects may play an essential role in β-cell defense against diabetogenic viruses. ISG20 (39, 40), viperin (*Rsad2*) (41, 42), and DDX60 (43) inhibit RNA viruses and some DNA viruses by multiple mechanisms including degradation of viral RNAs, termination of viral RNA synthesis, and enhancing RIG-I/MDA5 signaling.

**Figure S2.**
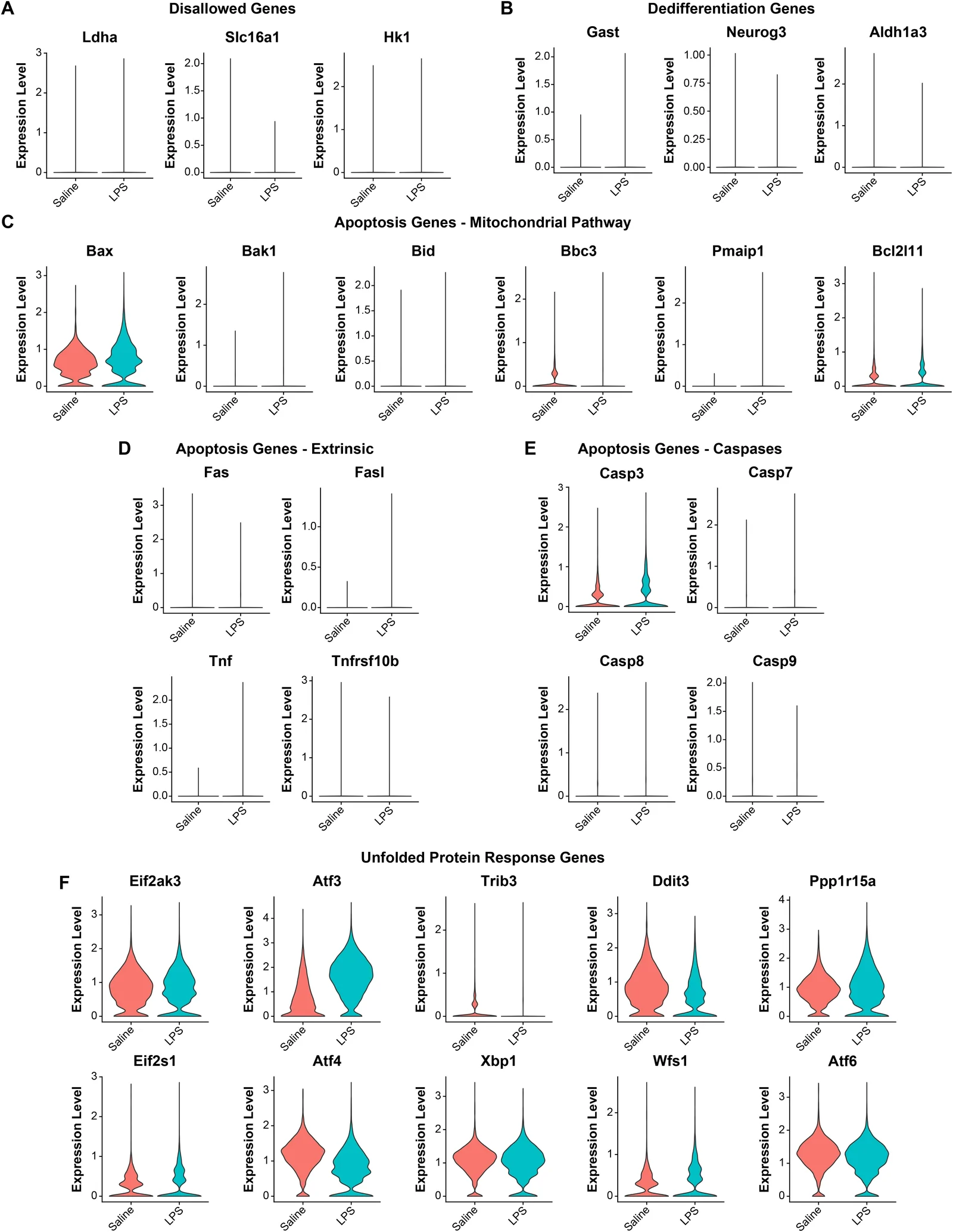
Expression of dedifferentiation, apoptotic, and stress genes in β-cells following LPS administration to mice. **(A)** Violin plots showing β-cell expression of disallowed, **(B)** dedifferentiation, **(C, D, E)** apoptotic, and **(F)** unfolded protein response (UPR) genes following LPS administration to mice.

The expression of genes “disallowed” in β-cells (*Ldha*, *Slc16a1*, and *Hk1*) or associated with β-cell dedifferentiation (*Gast*, *Neurog3*, *Aldh1a3*) was found to be unchanged in the β-cells from LPS-treated mice (Fig S2A and B). In addition, genes involved in apoptosis were unchanged (Fig S2C and E). A modest increase in *Casp3* expression was observed (log2FC = 0.42), however, this increase did not meet fold change criteria applied in this analysis (log2FC > 1; *P*-adj < 0.01). Genes that contribute to the unfolded protein response are not increased except for *Atf3* (Fig S2F), a transcription factor that has been reported to be proapoptotic or protective depending on context (44). Taken together, this analysis did not provide transcriptional evidence of β-cell damage or apoptosis 6 h after LPS administration.

**Figure S3.**
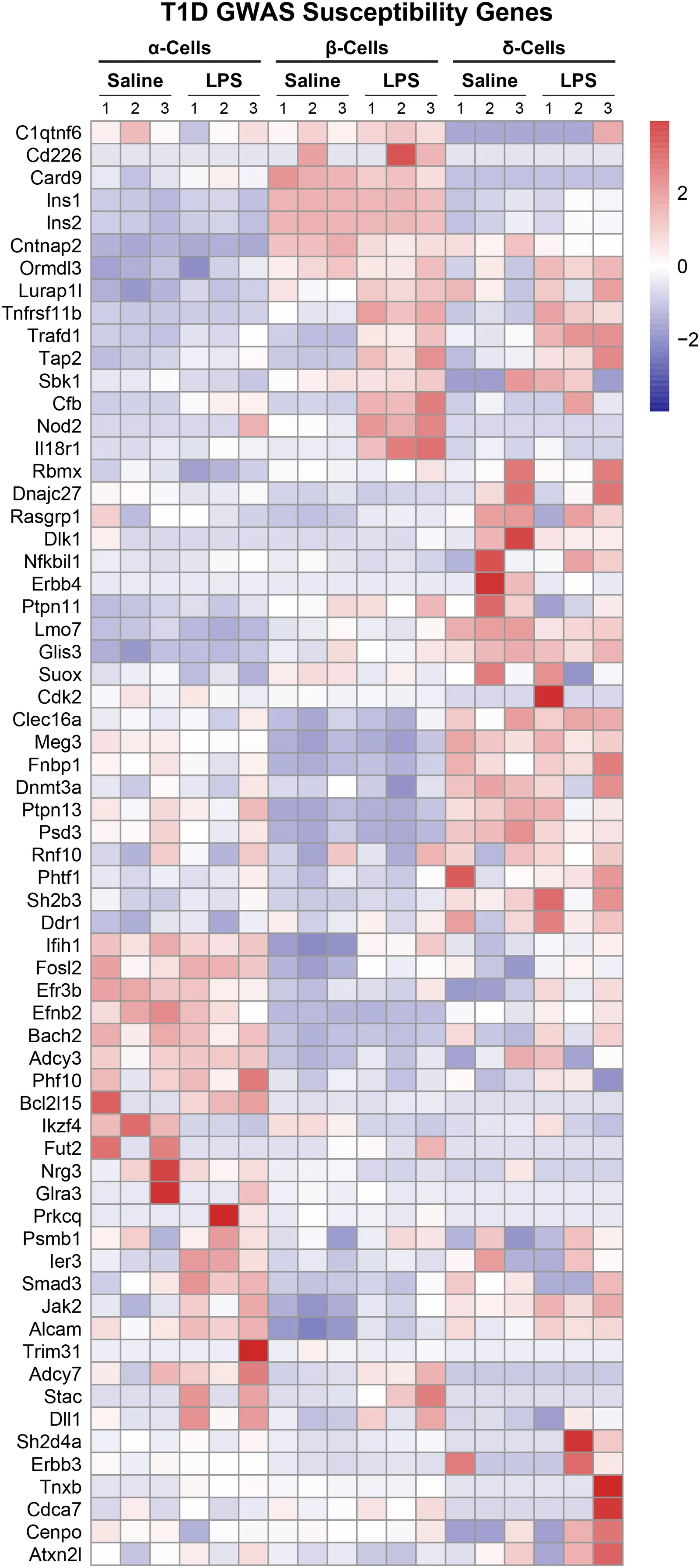
Expression of type 1 diabetes associated genes in islet endocrine cells following LPS administration to mice. Expression, normalized by row, of genes identified by genome wide association studies (GWAS) to be associated with T1D in α-, β-, and δ-cells.

Genome-wide association studies have identified genetic loci associated with T1D risk and it has been reported that >60 genes identified in these regions are expressed in β-cells (45). We investigated the expression of 64 of these genes in α-, β-, and δ-cells from mice after 6 h LPS exposure (Fig S3). Of the 64 genes analyzed in this study, 9 genes (*Il18r1*, *Ifih1*, *Tap2*, *Tnfrsf11b*, *Trafd1*, *Alcam*, *Fosl2*, *Cfb*, and *Jak2*) were significantly up-regulated (log2FC > 1, *P*-adj < 0.01) in β-cells after LPS exposure and the expression of 1 gene (*Cntnap2*) was decreased (log2FC < 1, *P*-adj < 0.01).

### Genes down-regulated in β-cells isolated from LPS-treated mice

**Figure 3.**
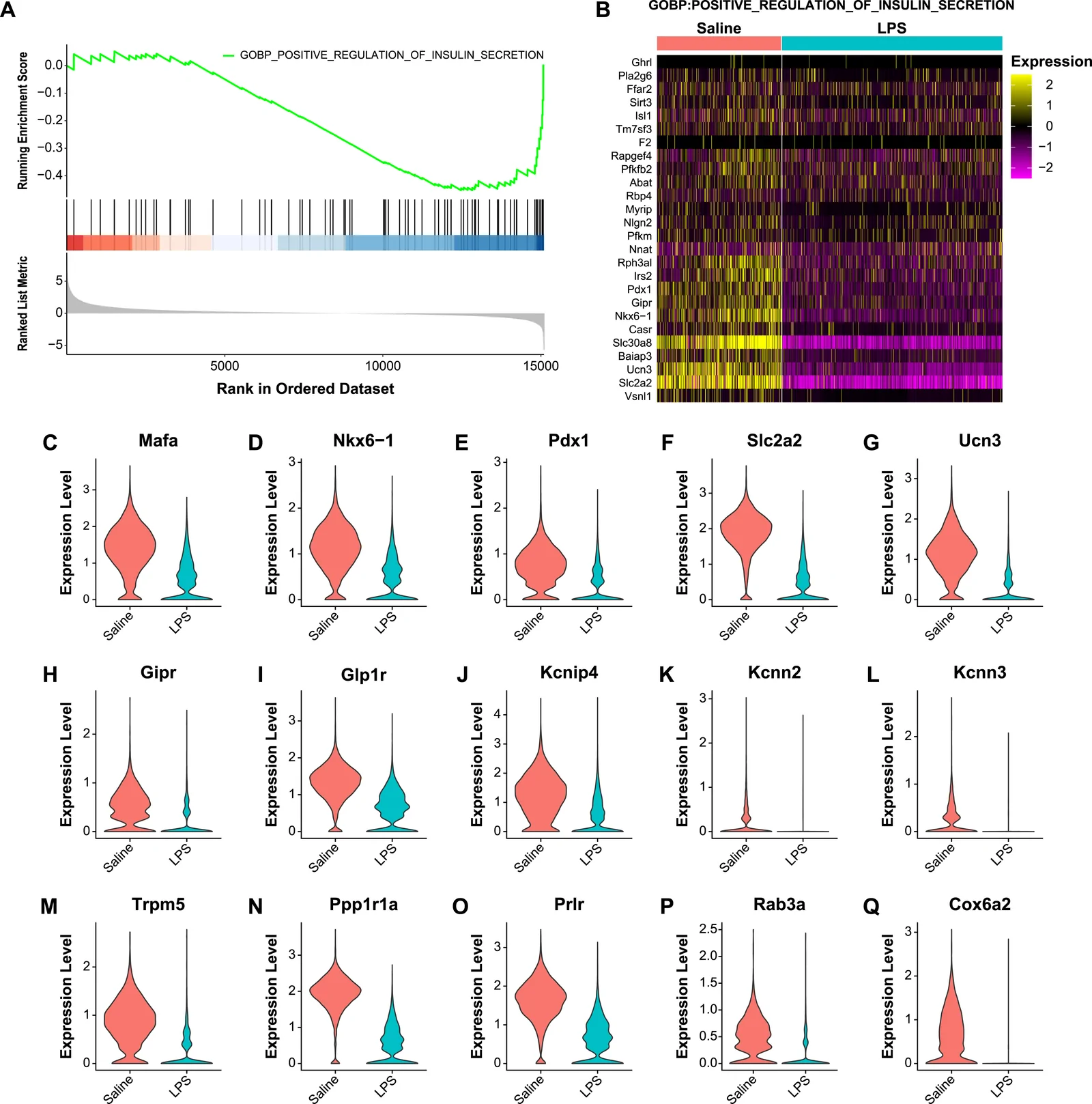
β-cell genes with reduced expression following LPS administration to mice. **(A)** Enrichment plot for the GO term “Positive Regulation of Insulin Secretion” from *MSigDB C5: Ontology Gene Set* curated by the BROAD Institute. **(B)** Heat map showing β-cell expression of genes annotated with the GO term “Positive Regulation of Insulin Secretion.” **(C, D, E, F, G)** Violin plots showing the expression level of genes in β-cells related to β-cell identity, **(H, I)** incretin hormone receptors, **(J, K, L)** ion channels, **(M, N, O, P)** insulin secretion, or **(Q)** apoptosis.

In addition to the 1,242 genes that were up-regulated in β-cells isolated from mice after LPS administration, expression of 527 genes was decreased with log2(FC) < 1 and *P*-adj < 0.01. Notable among these are genes involved in β-cell identity and function. Fig 3A shows the running sum for genes annotated with the term “Positive Regulation of Insulin Secretion” as curated by the BROAD Institute *MSigDB C5: Ontology Gene Set*. Overall, genes with this GO Term are significantly underrepresented in β-cells from mice treated with LPS (Fig 3B). In vitro studies using isolated islets have reported decreased expression of β-cell identity factors and genes involved in β-cell function following 6–12 h cytokine exposure (21, 46, 47). The gene and protein expression of several of these identity factors have recently been shown to transiently decrease in islets isolated from mice following LPS administration (31). Here, in addition to repression of β-cell identity factors (Fig 3C–G), we report attenuated expression of the incretin hormone receptors *Gipr* and *Glp1r* in vivo (Fig 3H and I). Genes encoding ion transporters that contribute to β-cell depolarization/repolarization and insulin secretion, such as *Kcnip4*, *Kcnn2*, *Kcnn3*, and *Trpm5*, are also repressed (Fig 3J–M). Consistent with reduced expression of genes from these broad categories related to insulin secretion, we also observe reduced expression of genes that promote β-cell function through other mechanisms including *Ppp1r1*, *Prlr*, and *Rab3a* (Fig 3N–P). The proteins encoded by these genes are essential for incretin-mediated amplification of insulin secretion (IPPR and PRLR) and enhance insulin granule docking and exocytosis (RAB3A). Interestingly, of all genes with reduced expression, the gene with the lowest false discovery rate (FDR) is *Cox6a2*, a subunit of mitochondrial complex IV reported to be proapoptotic (Fig 3Q) (48).

### Gene expression in α- and δ-cells isolated from LPS-treated mice

We have shown that many of the transcriptional responses stimulated by cytokine treatment of isolated islets are common among the individual endocrine cell populations of the islet (21, 22). To evaluate whether this occurs in vivo, α-, β-, and δ-cell populations were computationally isolated from our scRNAseq data set and changes in mRNA accumulation in response to LPS administration in α- and δ-cells were compared with changes observed in β-cells. Differential gene expression in each cell type was assessed using pseudobulk approaches to account for variability in the number of each cell type recovered. Compared with the 399 genes showing increased expression in β-cells in response to LPS, fewer genes were statistically increased in α-cells (237 genes) and δ-cells (14 genes). The lower number of genes showing differential expression is consistent with the increase in sampling noise and dispersion in samples derived from smaller cell populations. This results in reduced statistical power to detect differential gene expression and increased frequency of false negatives.

**Figure 4.**
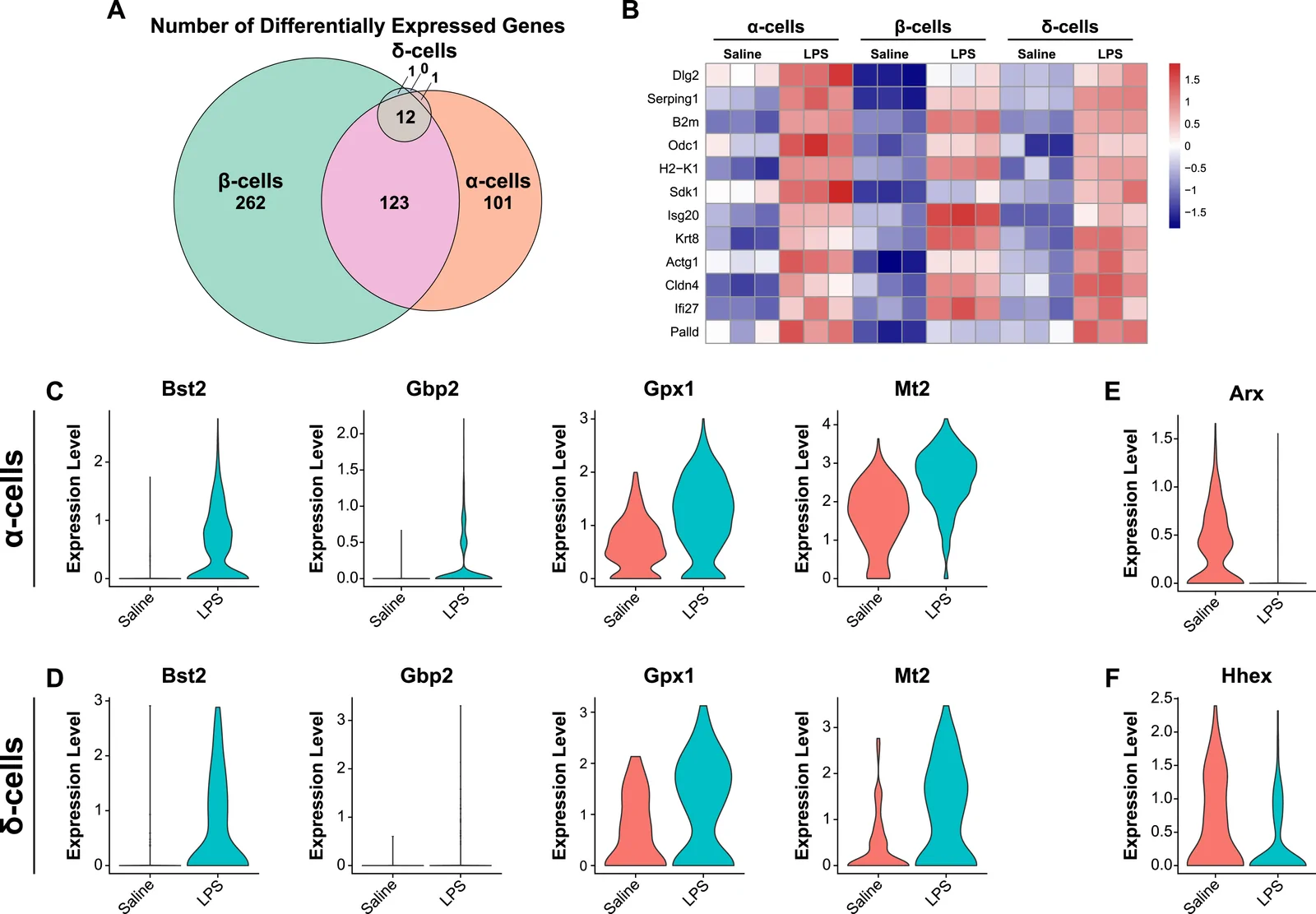
Islet endocrine cell gene expression following LPS administration to mice. **(A)** Euler plot showing the number of significantly up-regulated genes (log2FC > 1, *P*-adj < 0.01) in α-, β-, or δ-cells 6 h after LPS administration to mice. **(B)** Heat map showing expression of the 12 genes significantly up-regulated all three endocrine cell populations analyzed. **(C, D, E, F)** Violin plots showing expression of select genes in α- and δ-cells after LPS administration.

**Figure S4.**
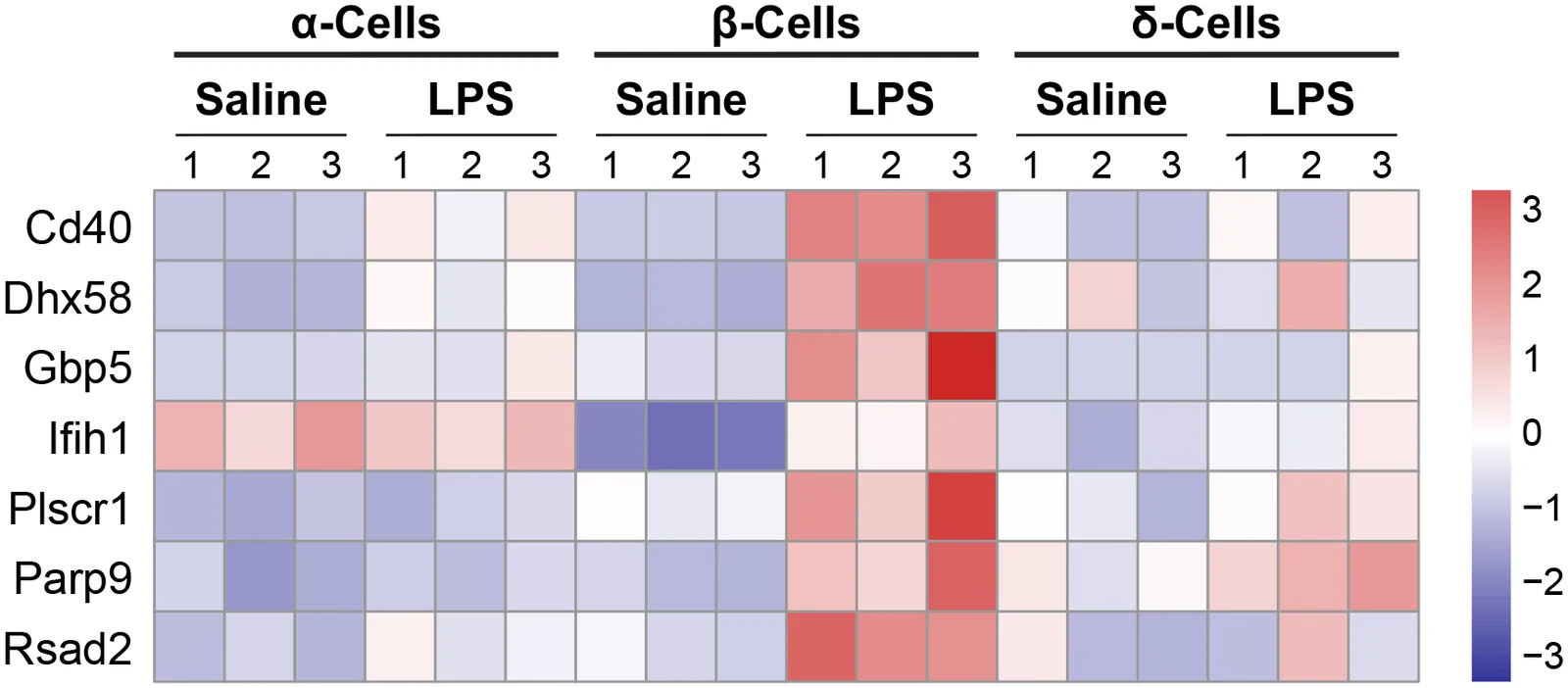
Genes up-regulated specifically in β-cells following LPS administration to mice. Expression, normalized by row, of genes up-regulated in β-cells, but not α- or δ-cells following LPS administration to mice.

Consistent with prior in vitro observations (21, 22), most of the genes up-regulated in α- and δ-cells are also up-regulated in β-cells, indicating a conserved response to endogenous cytokines among the endocrine cell populations of the pancreas (Fig 4A). There are 12 genes that are up-regulated in all three of the endocrine cell types analyzed here (Fig 4B). These genes are involved in antiviral defense (*Isg20* and *Ifi27*), maintenance of cell structure (*Dlg2*, *Sdk1*, *Krt8*, *Actg1*, *Cldn4*, and *Palld*), inhibition of complement (*Serping1*), and MHC I dependent antigen presentation (*B2m* and *H2-K1*). Additional genes such as the antiviral genes *Bst2* and *Gbp2* and the antioxidant genes *Gpx1* and *Mt2* are increased in β- and α-cells from mice treated with LPS, and are increased but lack the statistical power to achieve significance in δ-cells, likely due to the limited number of δ-cells recovered (Fig 4C and D). Although many transcriptional responses are shared among the endocrine cells analyzed here, there are some cell type specific differences in gene expression. For instance, several antiviral genes are up-regulated in β-cells, but not in either of the other endocrine cell types evaluated, suggesting some responses are unique to the β-cell (Fig S4).

In cultured islets, the cytokine dependent repression of identity genes is not limited to just β-cells (21, 22). We show that, in vivo, expression of the α-cell identity gene, *Arx*, is repressed (Fig 4E). Similarly, the δ-cell identity gene, *Hhex*, has reduced expression (Fig 4F) that does not achieve the threshold of significance (log2FC = −1.28, *P*-adj = 1) 6 h after LPS administration.

### Effects of LPS administration on islet endothelial cell gene expression

**Figure 5.**
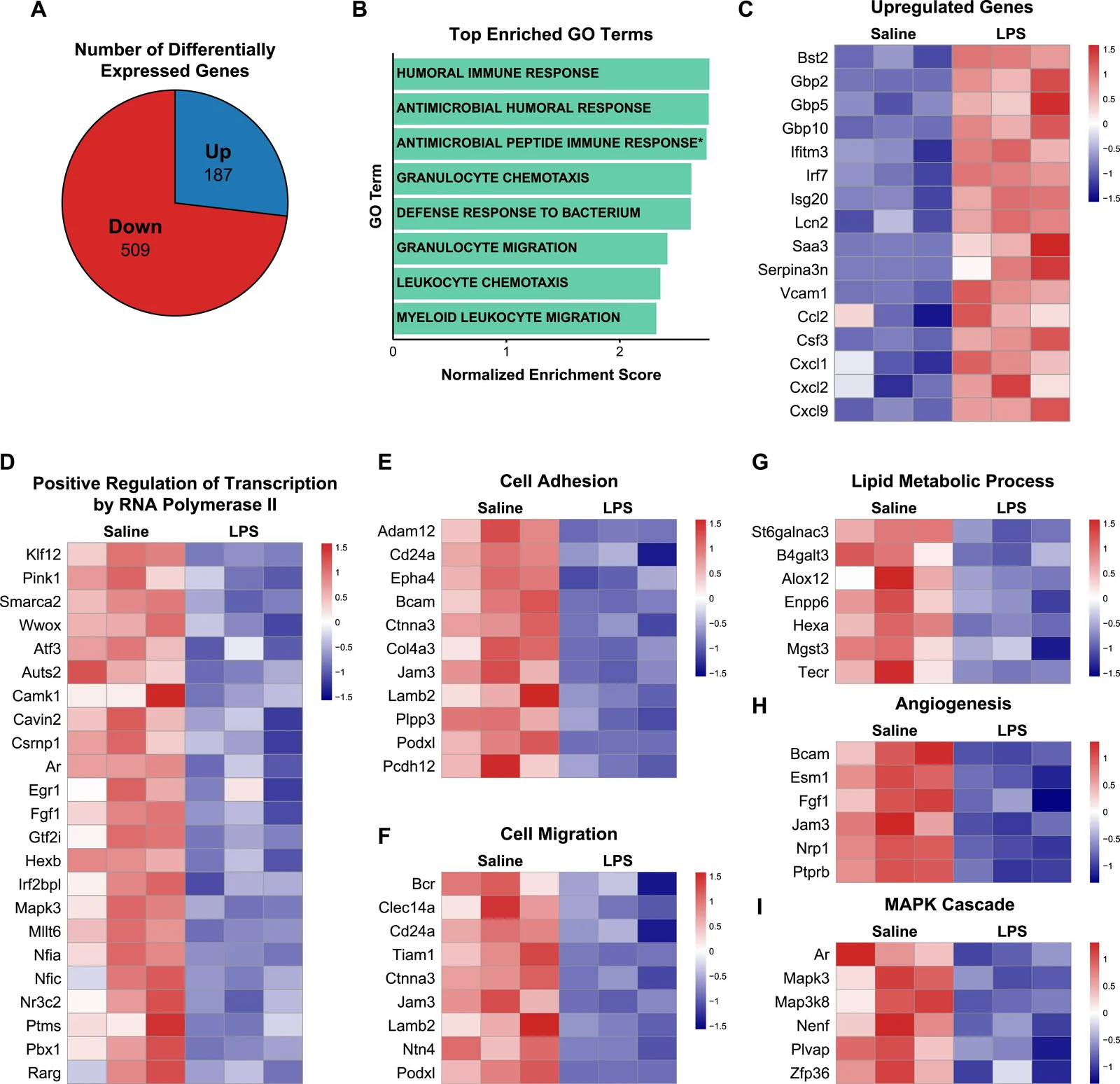
Endothelial cell gene expression following LPS administration to mice. **(A)** Pie chart showing the number of differentially expressed genes (|log2FC| > 1, *P*-adj < 0.01) in endothelial cells 6 h after LPS administration to mice. **(B)** Most highly-enriched GO terms in endothelial cells determined by gene set enrichment analysis (GSEA) using the *MSigDB C5: Ontology Gene Set* curated by the BROAD Institute. *The GO Term “Antimicrobial Humoral Immune Response Mediated By Antimicrobial Peptide” has been abbreviated as “Antimicrobial Peptide Immune Response” here. **(C)** Heat map showing the expression of the antiviral genes *Gbp2*, *Gbp5*, *Bst2*, and *Isg20* in endothelial cells after LPS administration to mice. **(D)** Heat maps showing the expression of genes repressed in endothelial cells annotated with the GO terms “Positive Regulation of Transcription by RNA Polymerase II,” **(E)** “Cell Adhesion,” **(F)** “Cell Migration,” **(G)** “Lipid Metabolic Process,” **(H)** “Angiogenesis,” or **(I)** “MAPK Cascade.”

Islet endothelial cells (IECs) participate in the development, differentiation, and function of pancreatic β-cells. IEC and β-cells are in frequent communication with each other and programmatic changes in one cell type can affect the other (49, 50). For this reason, we investigated the transcriptional responses in the endothelial cell population of our dataset. While the predominant transcriptional response in the endocrine cell population of islets isolated from LPS-treated mice was an increase in gene expression, in IEC only 27% (187/696) of the differentially expressed genes were increased (Fig 5A). The most highly enriched gene categories (Fig 5B) include immune and defense response genes, similar to β-cells, but with greater representation of humoral response and chemotaxis genes. Additionally, many of the antiviral genes with increased expression in the endocrine cell populations are also stimulated in IECs isolated from LPS treated mice. Overall, genes up-regulated in the endothelial cells (Fig 5C) reflect a cytokine-driven response signature with expression of interferon stimulated genes (ISG) and genes associated with antipathogen response.

**Figure S5.**
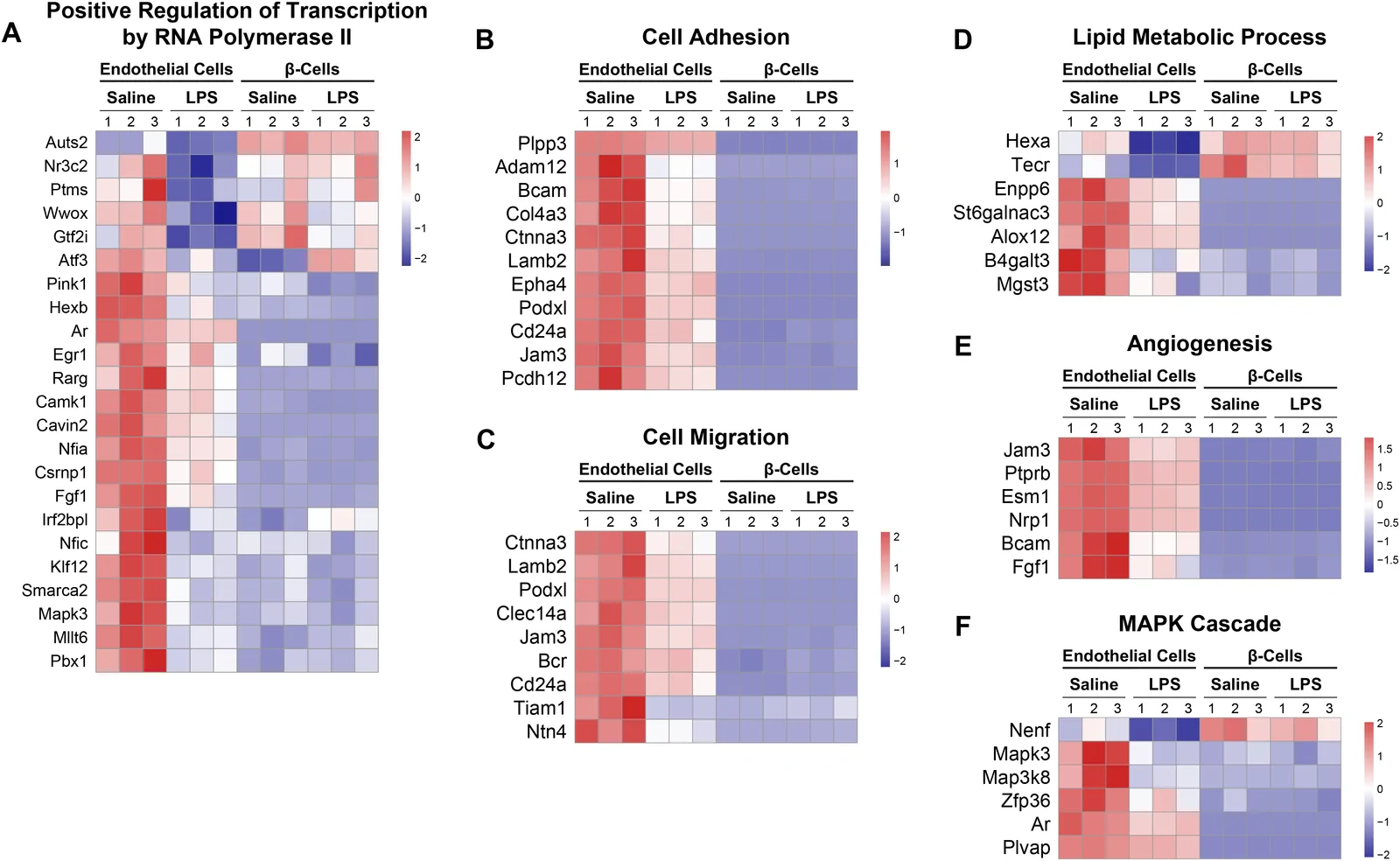
Endothelial and β-cell gene expression following LPS administration to mice. **(A)** Heat maps showing the expression of genes repressed in endothelial cells annotated with the GO terms “Positive Regulation of Transcription by RNA Polymerase II,” **(B)** “Cell Adhesion,” **(C)** “Cell Migration,” **(D)** “Lipid Metabolic Process,” **(E)** “Angiogenesis,” or **(F)** “MAPK Cascade.”

In contrast to primarily increased gene expression in islet endocrine cells following LPS administration to mice, the majority of differentially expressed genes in endothelial cells have reduced expression. Functional enrichment analysis of genes with decreased expression in IEC identified positive regulation of transcription by RNA polymerase II, cell migration, MAPK cascade, cell adhesion, angiogenesis, and lipid metabolic process as categories of genes that decreased in expression. The effects of LPS administration on genes with decreased expression that have been annotated with each of these GO Terms are shown in Fig 5D–I. Overall, these are not categories of genes that are universally repressed among all islet cell types analyzed in these studies, but are selective for IEC. As shown in Fig S5A–F, the expression profiles of these genes differ between endothelial cells and β-cells. Taken together, these data show that the response to innate immune activation of the islet vasculature differs from the endocrine compartment; repression of gene expression is more prevalent, and the biological pathways modified include reduced transcription, metabolism, growth, migration, and cell adhesion in IECs. The antipathogen response, however, is consistent between endothelial and endocrine cells.

### Effects of LPS administration on gene expression in islet-resident immune cells

**Figure 6.**
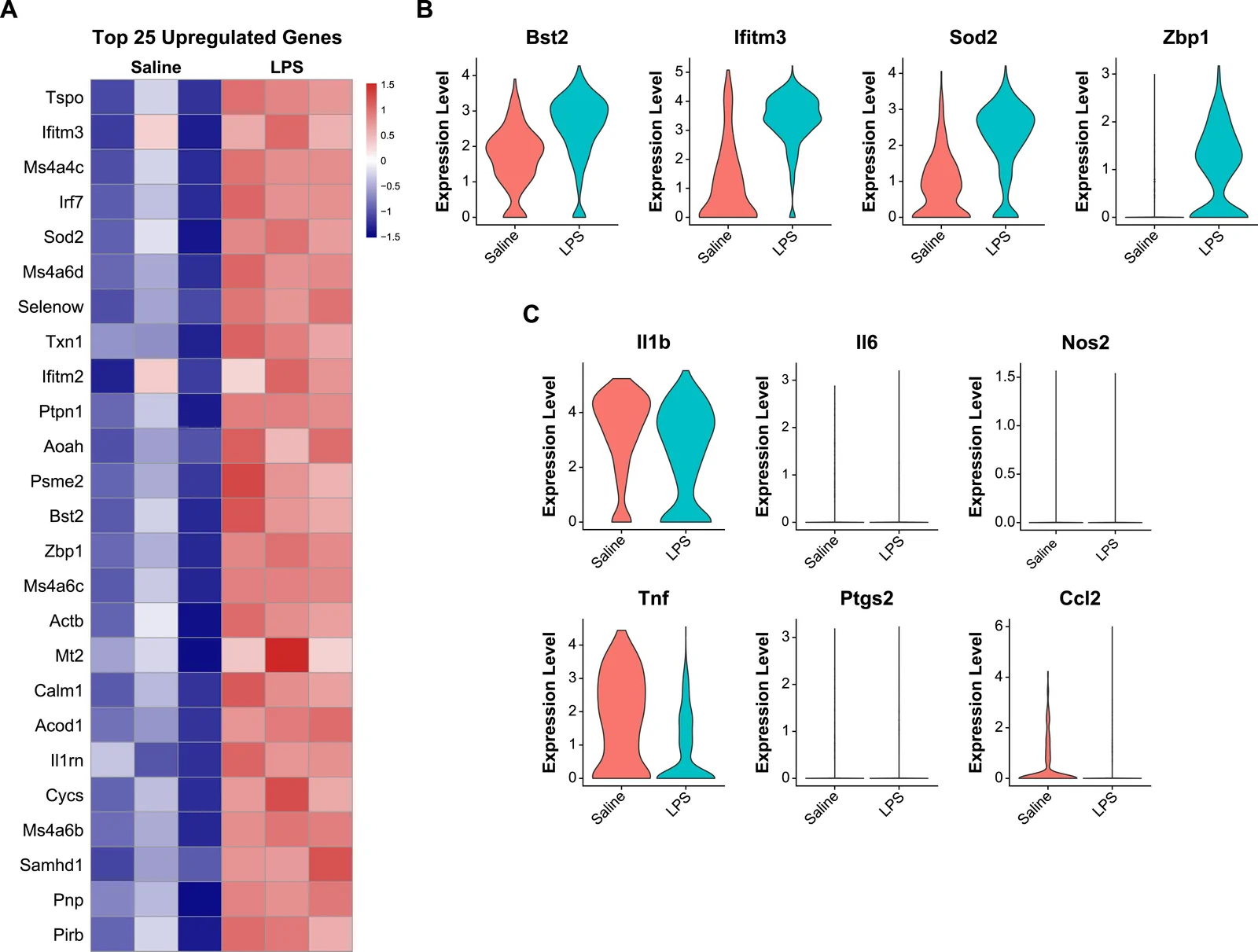
Non-lymphoid hematopoietic cell gene expression following LPS administration to mice. **(A)** Heat map showing the expression of the top 25 most highly enriched genes in non-lymphoid hematopoietic cells 6 h after LPS administration to mice. **(B)** Violin plots showing the expression of select defense genes and **(C)** classical markers of activation in non-lymphoid hematopoietic cells.

To evaluate the effects of innate immune activation on islet-resident immune cells, we computationally isolated the non-lymphoid hematopoietic cells (cells that express *Ptprc* [CD45] but not *Trbc* [T-lymphocytes] or *Ighm* [B-lymphocytes]), which are primarily islet resident macrophages. This population also displays enriched expression of *Cd68*, *Itgam*, *Adgre1*, and *Cxcl16*, supporting the presence of circulating monocytes and/or dendritic cells. The top 25 most highly enriched genes in this population of non-lymphoid hematopoietic cells are shown in Fig 6A and, unsurprisingly, include immune response genes such as *Bst2*, *Sod2*, *Ifitm3*, and *Zbp1* whose protein products are known to contribute to defense responses to pathogens (Fig 6B). Although these non-lymphoid hematopoietic cells display clear evidence of responding to stimuli following LPS administration, there is not a clear enrichment of classical markers of activation that one might expect following LPS stimulation of macrophages/monocytes/dendritic cells. Specifically, Il*1b*, *Il6*, *Nos2*, *Tnf*, *Ptgs2*, or *Ccl2* are not increased in this cell population following LPS administration that stimulates systemic innate immune activation (Fig 6C). These findings were surprising, as we have previously shown that in vitro treatment of rat, mouse, and human islets with PAMPs, such as LPS and poly IC, results in islet-resident macrophage activation and release of IL-1 locally to levels sufficient to inhibit β-cell function (6, 51, 52). Given that these resident non-lymphoid hematopoietic cells found in islets respond to LPS administration in vivo with increases in immune response genes, but do not increase the expression of genes associated with classical activation, we hypothesize that these cells are: (i) being exposed to cytokines, but not LPS, perhaps due to unique islet microvasculature or (ii) do not take on a proinflammatory phenotype in response to LPS stimulation, perhaps in an effort to minimize islet inflammation.

### IL-1 is a primary mediator of changes in β-cell gene expression following innate immune activation in mice

IL-1 is a key mediator of the inhibitory and destructive actions of cytokines on GSIS and islet integrity following in vitro treatment (2, 3). The damaging actions are mediated by IL-1-induced expression of iNOS and production of NO by the β-cells (8, 9, 10, 11). RNA-sequencing of islets cultured with cytokines in vitro indicate that IL-1 has a greater influence on β-cell gene expression than IFN-γ and that IL-1, independently of IFN-γ, up-regulates antiviral genes in β-cells (21). We have recently shown that LPS administration to mice increases serum cytokine levels and results in changes in islet gene and protein expression that are dependent on IL-1 signaling in the β-cell (31). Additionally, LPS exposure leads to a transient reduction in blood glucose concentration in mice that is dependent on IL-1 (53).

**Figure 7.**
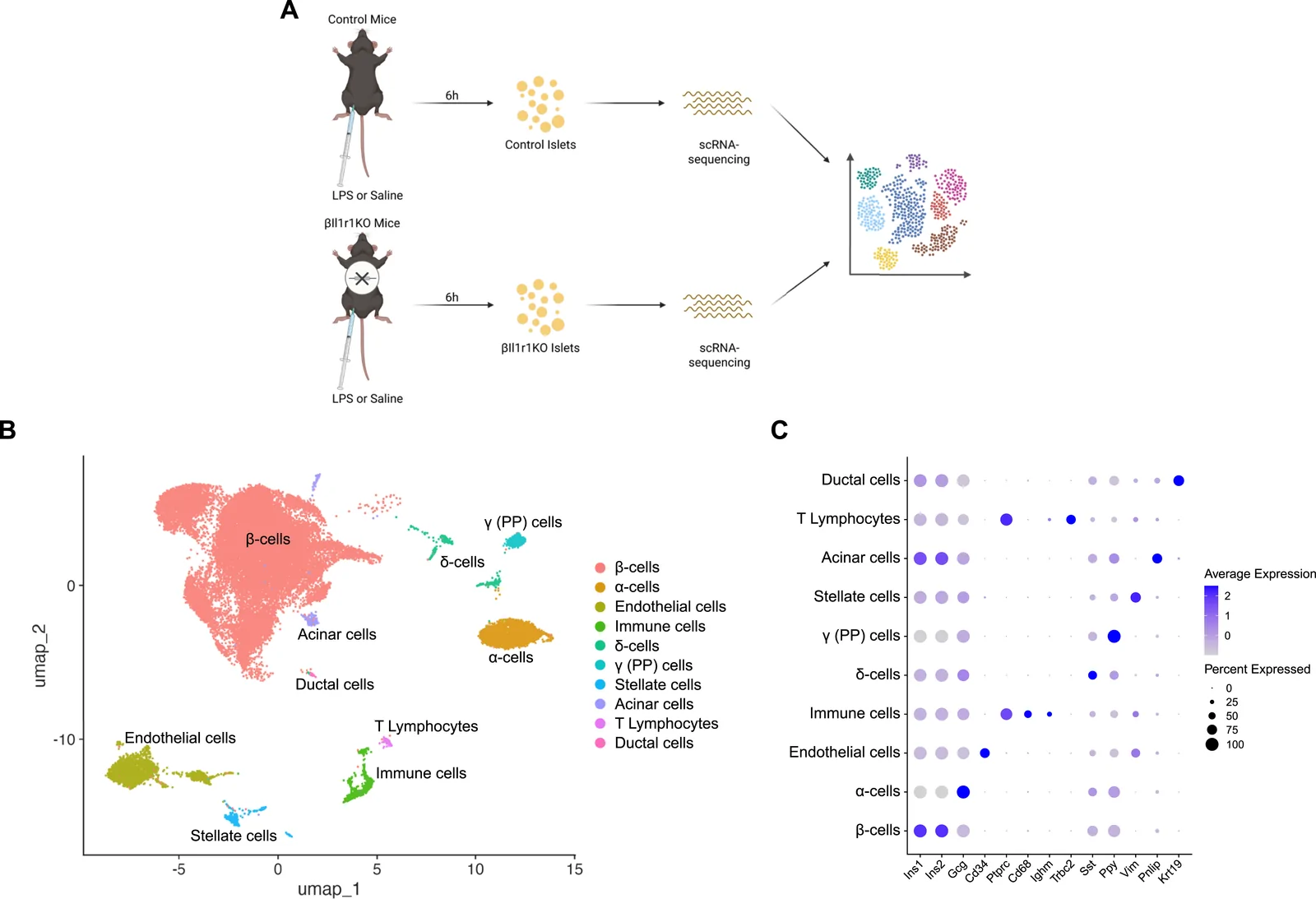
Single-cell RNA sequencing of islet cells following LPS administration to βIl1r1KO mice. **(A)** Experimental design schematic. **(B)** Uniform Manifold Approximation and Projection (UMAP) plot color-coded by cell identity based on characteristic gene expression. The “Immune Cell” population refers to cells that express *Ptprc* (CD45) excluding T-lymphocytes, which are identified separately. **(C)** Dot plot indicating expression of select marker genes in each cell population.

To more comprehensively investigate the transcriptomic consequences of IL-1 signaling in β-cells and determine the transcriptional responses for which IL-1 signaling is necessary, we administered LPS (0.33 mg/kg) or endotoxin-free physiological saline by IP injection to mice with a β-cell-specific deletion of the IL-1 signaling receptor (βIl1r1KO) and littermate control animals (31). Islets were isolated 6 h later and immediately subjected to scRNA-seq (Fig 7A). Cells from eight samples (two biological replicates per genotype-condition) were visualized with a UMAP plot (Fig 7B) and cell identity was assigned to each cluster based on expression of characteristic genes (Fig 7C).

**Figure S6.**
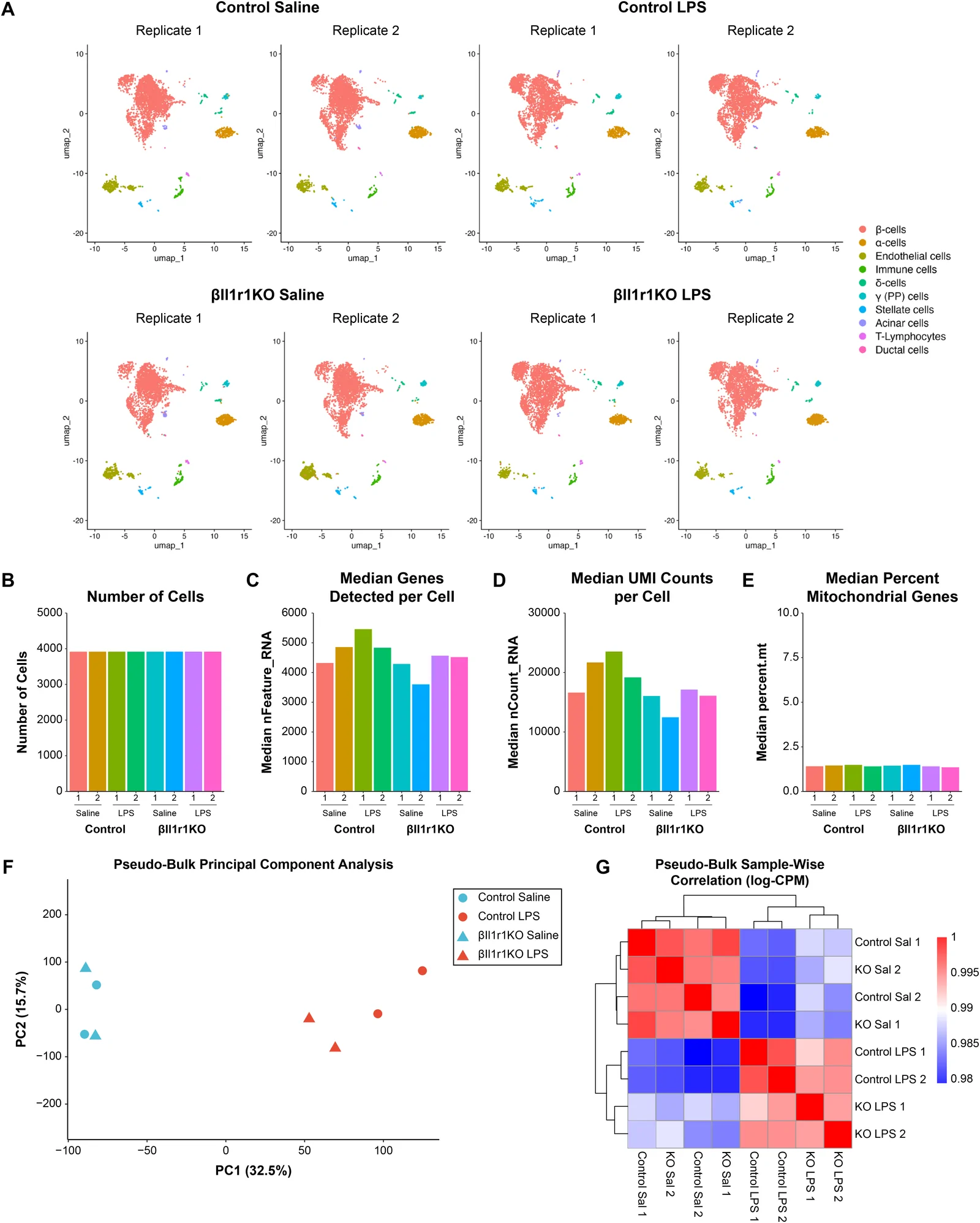
βIl1r1KO scRNA-seq replicate consistency. **(A)** Uniform Manifold Approximation and Projection (UMAP) plots split by each biological replicate and color-coded by cell identity. **(B)** The number of cells, **(C)** median number of detected genes (nFeature_RNA), **(D)** median unique molecular identifier (UMI) counts (nCount_RNA), and **(E)** median percent mitochondrial genes (percent.mt) per sample. **(F)** Pseudo-bulk expression profiles were generated by summing raw UMI counts, normalizing to counts-per-million (CPM), and applying log1p transform. Principle component analysis of pseudo-bulk samples is shown. **(G)** Sample-wise Pearson correlation coefficients with dendrogram.

To ensure variability across biological replicates and treatment conditions was not concealed by combining replicates in downstream analyses, replicate consistency was evaluated. We observed a high level of consistency across biological replicates as shown in Fig S6A–G. To ensure consistency of statistical comparisons made across all four conditions (βIl1r1KO saline, βIl1r1KO LPS, control saline, and control LPS), cells were randomly sampled from each experimental replicate such that each total number of cells was equal across all replicates (3,918 cells/replicate).

### IL-1 is a key mediator of β-cell responses to LPS administration and innate immune activation in mice

As shown in Figs 1, 2, 3, 4, 5, and 6, administration of LPS to mice significantly modifies gene expression in islet endocrine and endothelial cells but does not activate non-lymphoid hematopoietic cells in a classical manner (*Il1b* and *Tnf* levels are not increased, Fig 6C). These findings, and recent studies by our group (31), support our hypothesis that LPS administration stimulates innate immune activation and increases the levels of serum cytokines resulting in cytokine-mediated changes in gene expression in β-cells. To determine the importance of IL-1 in regulating the changes in β-cell gene expression, islets were harvested 6-h post LPS administration to βIl1r1KO and littermate control mice, dispersed into individual cells, and subjected scRNA-sequencing.

**Figure 8.**
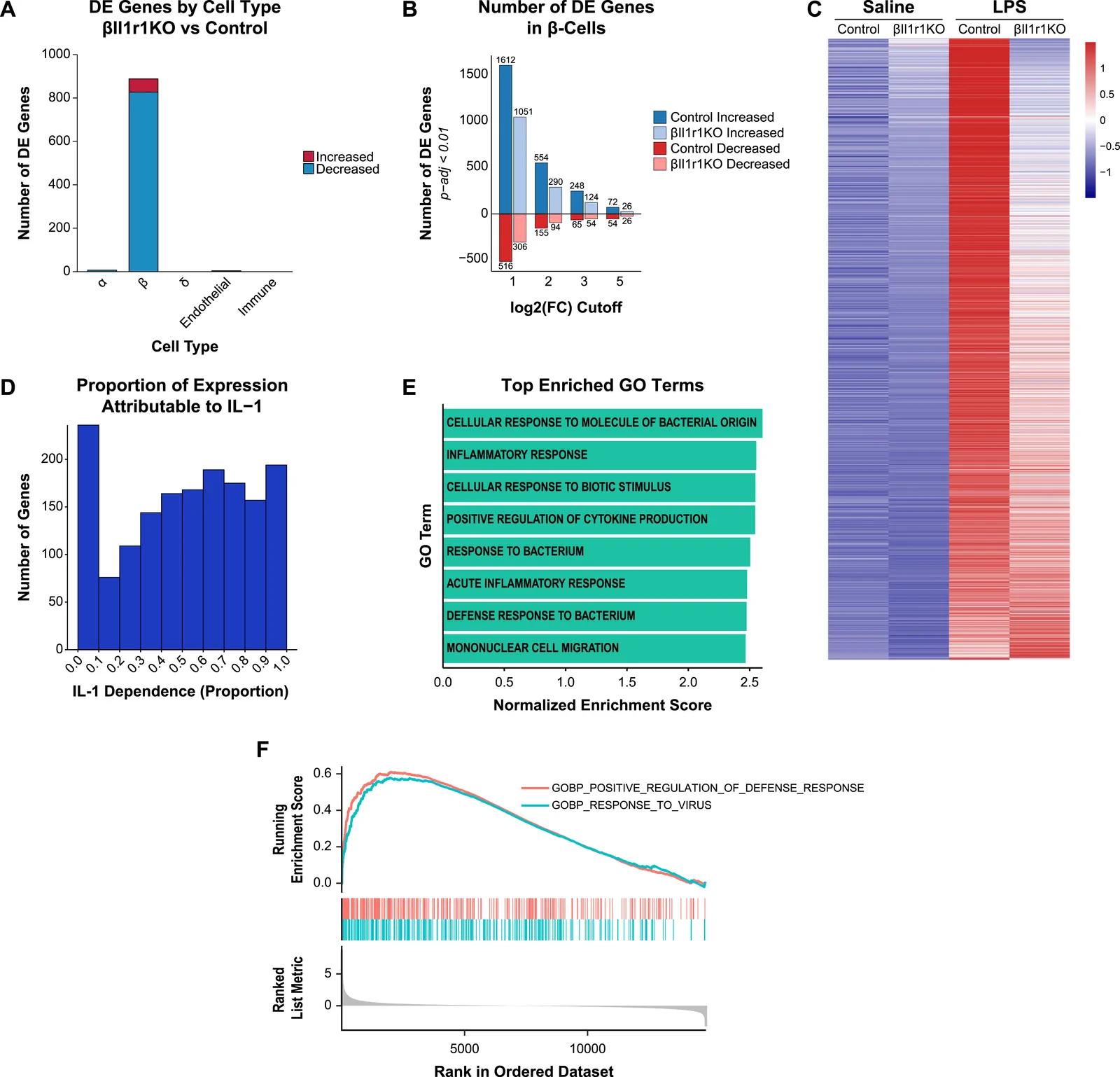
IL-1-dependent gene expression following LPS administration to mice. **(A)** Number of differentially expressed genes (|log2FC| > 1, *P*-adj < 0.01) (LPS treated βIl1r1KO mice versus littermate control mice). Genes with reduced expression in the absence of IL-1R are denoted as “decreased.” **(B)** Number of differentially expressed genes in β-cells (LPS versus saline) from βIl1r1KO or littermate control animals with *P*-adj < 0.01 at various log2FC thresholds. **(C)** Heat map showing the expression in each condition of the 1,612 genes up-regulated in control animals administered LPS versus saline. **(D)** For each gene up-regulated in β-cells from LPS-treated control animals, the proportion of gene stimulation dependent on IL-1R was calculated. The distribution of IL-1 dependence is shown in a histogram. Genes with IL-1 dependence <0 or >1 are included with the 0–0.1 or 0.9–1.0 bins, respectively. **(E)** Most highly-enriched GO terms for IL-1 dependent genes in β-cells (βIl1r1KO-LPS versus Control-LPS) determined by gene set enrichment analysis (GSEA) using the *MSigDB C5: Ontology Gene Set* curated by the BROAD Institute. **(F)** Enrichment plot for the GO terms “Positive Regulation of Defense Response” (NES = 2.37, *P*-adj = 9.93e−09) and “Response to Virus” (NES = 2.24, *P*-adj = 9.93e−09).

Direct comparison of gene expression in β-cells from βIl1r1KO or control mice treated with LPS identified 888 IL-1 dependent genes (log2FC > 1, *P*-adj < 0.01) as shown in Fig 8A. Of these, 827 genes had decreased expression in the IL-1R-deficient β-cells as compared with control β-cells from LPS-treated mice indicating that IL-1 contributes to increased expression of these genes. Interestingly, although other cell populations in the islet respond to cytokines and paracrine signals from nearby β-cells, deletion of IL-1R from the β-cell almost exclusively affects gene expression in the β-cell (Fig 8A). Although paracrine signaling is known to regulate function in islets, these findings indicate that IL-1 does not initiate paracrine signaling from β-cells to other islet endocrine or non-endocrine cell populations to alter gene expression.

To evaluate the effect of IL-1R deficiency on β-cell gene expression, the number of genes in βIl1r1KO versus littermate control β-cells in response to LPS with *P*-adj < 0.01 and log2FC of at least 1.0–5.0 was determined (Fig 8B). In β-cells from control animals, 1,612 genes met the statistical threshold of log2FC > 1.0. In the Il1r1KO β-cells, only 1,051 genes met these same criteria, 35% fewer genes than when IL-1R is present. Furthermore, this relative difference is even more dramatic with more stringent fold change threshold. At a log2FC > 5.0, 64% fewer genes meet the differential expression threshold in IL-1R deficient β-cells than control β-cells. In addition to differences in up-regulated genes, 41% fewer genes were repressed following LPS administration in β-cells from Il1r1KO mice than control mice (516 versus 306 genes; log2FC < −1.0).

For each of the genes up-regulated following LPS treatment of control mice, expression level may be dependent on IL-1, independent of IL-1, or modulated by the presence of IL-1. A heat map showing the expression levels of genes up-regulated in control mice following LPS administration shows that IL-1 modulates the expression of the majority of these genes (Fig 8C). To quantitatively determine the magnitude of IL-1 dependence for each gene up-regulated following LPS exposure, the difference in post-LPS gene induction between β-cells with and without IL-1R was calculated for each of the 1,612 genes up-regulated (log2FC > 1.0 and *P*-adj < 0.01) in the control animals following LPS administration. A histogram depicting the IL-1 dependence of each gene is shown in Fig 8D. As expected, the inducible expression of over 200 genes is predominantly independent of IL-1 signaling. One example is *Bst2*, which is less than 10% dependent on the presence of IL-1R. Alternatively, the expression of a large number of genes, including *Nos2*, is greater than 90% dependent on IL-1 signaling. Consistent with the qualitative expression shown in Fig 8B, IL-1 modulates the expression of the majority of the genes up-regulated following LPS administration. Approximately three quarters of the genes are at least 30% dependent on IL-1R, and 194 differentially expressed β-cell genes are almost entirely dependent on the presence of IL-1R (the proportion of expression attributable to IL-1R > 0.9). Importantly, this occurs in the presence of other soluble mediators like type I and type II interferon, TNF-α, and others.

Gene set enrichment analysis was performed using differential gene expression between LPS-treated wild type control and βIl1r1KO mice to determine which GO terms are enriched by IL-1 signaling. The eight most highly enriched categories of genes are shown in Fig 8E and are predominantly antibacterial and immune response genes, suggesting the β-cell responses initiated by IL-1 are physiologic and protective. Although antiviral and general defense response pathways were not observed within the top eight terms, IL-1 does initiate these transcriptional responses as the terms “Positive Regulation Of Defense Response” (NES = 2.37) and “Response To Virus” (NES = 2.24) are highly enriched (Fig 8F).

### IL-1 requirements for specific genes/gene categories in β-cells

**Figure 9.**
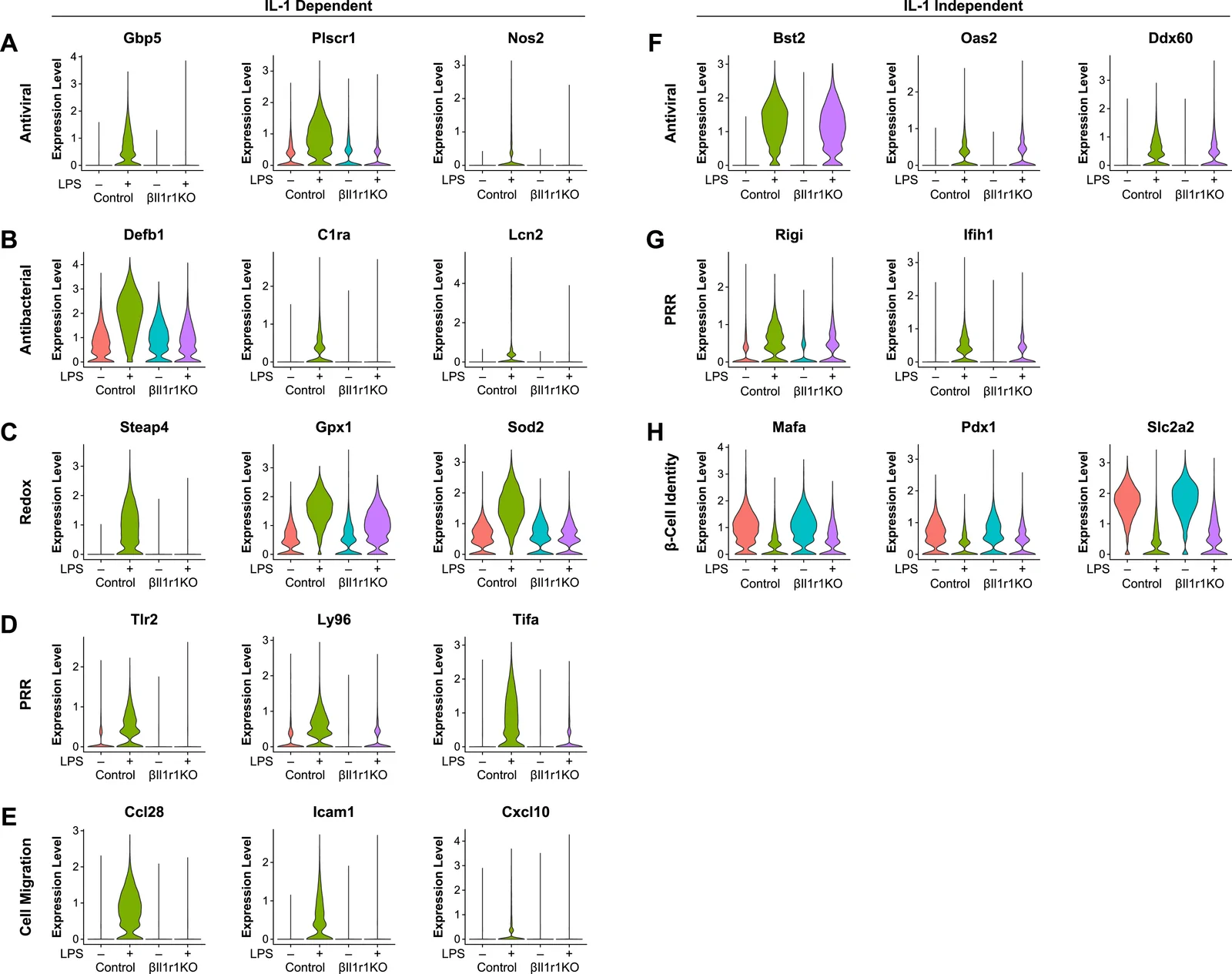
IL-1-dependent gene expression in β-cells. Violin plots showing the expression in β-cells of select IL-1-dependent genes that encode proteins reported to be involved with various functions including: **(A)** antiviral, **(B)** antibacterial, **(C)** redox, **(D)** pattern recognition receptors (PRR), **(E)** cell migration. Violin plots showing the expression in β-cells of select IL-1-independent genes that encode proteins reported to be involved with various functions: **(F)** antiviral, **(G)** PRR, **(H)** β-cell identity.

Genes with a variety of functions, that are thought to play physiologically relevant protective roles, are up-regulated in β-cells following LPS administration in an IL-1-dependent manner (Fig 9). Genes whose products are functionally antiviral (*Gbp5*, *Plscr1*, and *Nos2*) and antibacterial (*Defb1*, *C1ra*, and *Lcn2*) are up-regulated in β-cells that express IL-1R, but not in β-cells that lack this signaling receptor (Fig 9A and B). Genes whose products directly interfere with pathogens, support host response to infection, or encode redox proteins and pattern recognition receptors (PRRs) are enhanced in an IL-1-dependent manner in β-cells following LPS administration. Additional redox active genes induced in an IL-1-dependent manner include *Steap4*, *Gpx1*, and *Sod2*. The products of these genes may function to protect β-cells from potential reactive oxygen species (ROS) produced by nearby innate immune cells (Fig 9C) while pattern recognition receptors including *Tlr2*, *Ly96*, and *Tifa* contribute to host-defense and PAMP recognition (Fig 9D). Expression of genes that encode proteins known to contribute to immune cell migration such as *Ccl28*, *Icam1*, and *Cxcl10 *are also enhanced in β-cells that express IL-1R (Fig 9E).

Not all of the genes with enhanced expression in β-cells in response to LPS administration are IL-1-dependent. We have highlighted a number of genes that encode antiviral proteins (*Bst2*, *Oas2*, and *Ddx60*) or pattern recognition receptors (*Rigi* and *Ifih1*) that are expressed in β-cells in an IL-1-independent manner in mice administered LPS (Fig 9F and G). We believe that this highlights the presence of multiple signaling cascades that increase the expression of genes encoding proteins in defense response pathways that may contribute to the survival of this essential cell type. Many of these genes are known to be responsive to IFNs and TNF-α. Additionally, the repression of β-cell identity genes, which occurs in response to IL-1 in culture (21, 22, 23), does not require the presence of IL-1 in vivo (Fig 9H) and is likely mediated by other cytokines such as TNF-α or type I interferon (31).

### IL-1 inhibits picornavirus mRNA accumulation in islets

**Figure 10.**
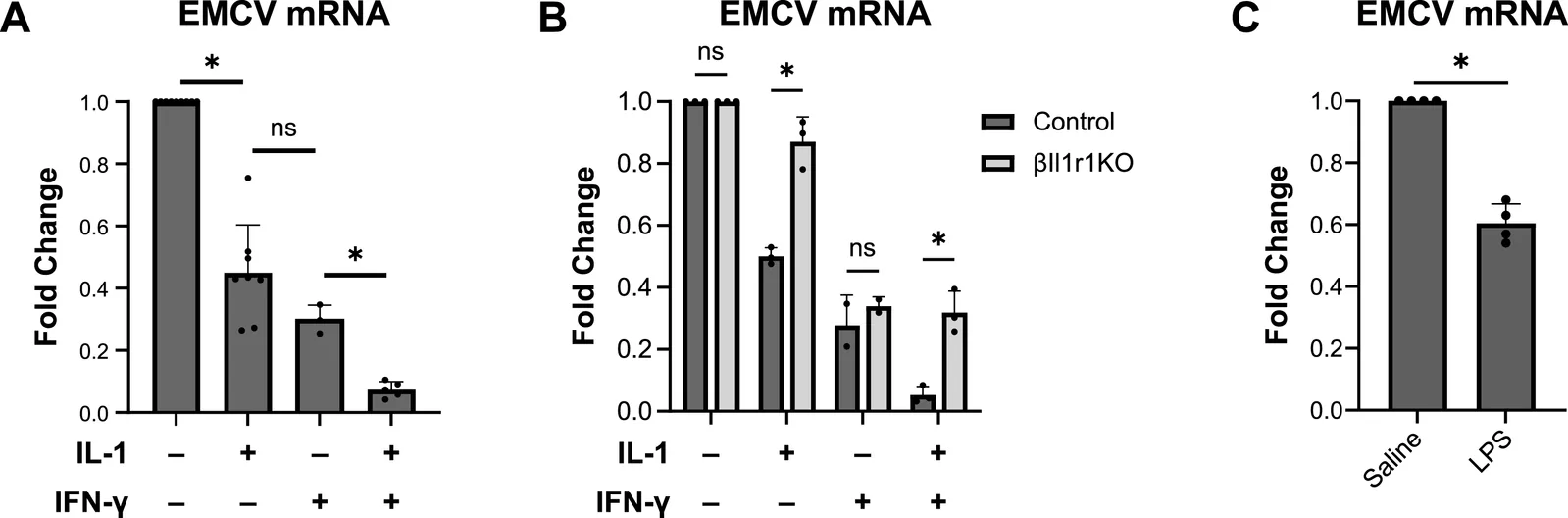
Antiviral effects of IL-1 in β-cells. **(A)** Islets from C57BL/6J mice or **(B)** βIl1r1KO and littermate control mice were pretreated with IL-1 (10 U/ml) and/or IFN-γ (150 U/ml) for 6 h before 12 h EMCV infection. Viral mRNA (EMCV VP1) is shown. **(C)** Islets isolated 6 h after i.p. administration of LPS to C57BL/6J mice were infected with EMCV for 12 h and viral mRNA (EMCV VP1) was quantified. Results are average ± SD of 3 to 8 independent experiments. Statistical significance calculated by two-way ANOVA (*P < 0.05 versus untreated control; ns = not significant) is indicated.

To assess the functional effect of cytokine exposure on viral replication, islets isolated from C57BL/6J mice were treated for 6 h with or without IL-1 and/or IFN-γ and then infected with 5 MOI of the diabetogenic picornavirus encephalomyocarditis virus (EMCV). As shown in Fig 10A, EMCV mRNA accumulation was reduced by >50% in islets treated with IL-1 (10 U/ml) or IFN-γ (150 U/ml) alone and by >90% in islets treated with a combination of both cytokines. Although IL-1 is not frequently described as a cytokine with potent antiviral activity, in islets, IL-1 inhibited viral replication as effectively as the more canonically antiviral cytokine, IFN-γ. The antiviral effects of IL-1 in the islet are mediated directly by IL-1 signaling in the β-cell. The inhibition of EMCV replication observed in response to IL-1 is lost in islets from βIl1r1KO mice, while the depletion of the IL-1R does not modify the antiviral actions of IFN-γ (Fig 10B). To test whether susceptibility to infection is reduced in islets following exposure to endogenously produced cytokines, islets isolated 6 h after LPS administration were immediately infected with EMCV and viral mRNA was then measured 12 h later. As shown in Fig 10C, viral mRNA accumulation is reduced by 40% in islets isolated from LPS-treated mice compared with islets isolated from saline-treated control animals. These findings support a functional role for cytokine signaling in the islet that enhances β-cell resistance to infection.

## Discussion

Over 40 years ago, Mandrup-Poulsen et. al. showed that incubation of isolated rodent and human islets with cytokines results in an inhibition of insulin secretion and loss of viability (1, 2). IL-1 was identified as the primary mediator of this damage and its actions are enhanced by IFN-γ and TNF-α. Based on the results of these and other early studies, it has been hypothesized that cytokines may directly cause β-cell death and dysfunction that contributes to the onset of type 1 diabetes. Yet, to date, a therapeutic strategy that targets or disrupts the actions of cytokines on β-cells that reduces the incidence of or prevents autoimmune diabetes has yet to be developed. In fact, treatment of NOD mice with molecular PAMPs or cytokines prevents diabetes development (54, 55, 56, 57), and NOD mice-deficient in cytokine signaling receptors develop diabetes (58, 59). We were one of the first groups to identify NO, produced following cytokine stimulated iNOS expression, as the primary mediator of the inhibitory actions of cytokines on insulin secretion and oxidative mitochondrial metabolism, yet despite significant effort by numerous groups dedicated to studying this effect, NOS inhibition does not delay the onset of diabetes in the NOD mouse (60).

More recently, studies have provided evidence that there are physiologic roles for cytokine signaling (61) and NO production in the islet (21, 22, 23, 28). In vitro studies using isolated islets and insulinoma cells have shown that NO inhibits apoptosis (28, 29), attenuates picornavirus replication (24, 25, 26), and stimulates the expression of protective DNA damage repair genes in β-cells (27, 28). Also, the actions of cytokines and NO are reversible, either by washing to remove the cytokine or inhibiting iNOS in the presence of cytokines (12, 13, 14). Consistent with physiological roles for cytokine signaling are the rapid and early increases in the expression of antiviral and antibacterial genes in all endocrine cell types within the islet following a short 6 h IL-1 incubation in culture (21). Importantly, there was no evidence of apoptotic gene or ER-stress gene expression under these conditions. In contrast, scRNA-sequencing of islets cultured with cytokines in vitro for prolonged periods of time, up to 48 h, display an increase in the expression of genes associated with cell stress and inflammation (16, 17, 19). In vitro studies of cytokine treated islets, however, do not fully capture the dynamic fluctuations in serum cytokines, blood glucose levels, presence of hormones, regulation of blood flow, and other small molecules to which islets are exposed in vivo. To gain more insight to this complexity, scRNA-seq has been performed on islets obtained from human donors with or without T1D (62, 63); however, it is not possible to determine if observed transcriptional profiles in these studies contribute to, or are the result of, disease onset. In the current study, we use a well-defined mouse model to capture islet cell responses to endogenously produced cytokines and obtain an unbiased transcriptomic profile for individual cell populations within the islet.

For these studies, islets were isolated 6-h post LPS administration to mice, dispersed into individual cells, and scRNA-seq was performed. Insulin-expressing β-cells were computationally isolated and gene set enrichment analysis was performed. The most highly enriched gene categories identified in β-cells included those related to defense response to pathogens (viral and bacterial), and many of the genes with increased expression from these categories were genes we identified in β-cells in response to a 6 h in vitro exposure to IL-1 and IFN-γ (21). We interpret this profile as an indication that β-cells are initiating a defense protocol in response to the rapidly disseminated soluble mediators produced by immune cells in response to LPS administration. We hypothesize that this is a protective response that enhances cellular defense, in case the β-cell is confronted with a pathogen. In support of this hypothesis, we show that IL-1, alone or in combination with IFN-γ, attenuates EMCV mRNA accumulation in islets. In addition to the expression of antiviral genes, the expression of genes related to immune cell trafficking (e.g., *Cxcl10*, *Icam1*) and MHC class I (e.g., *B2m*, and *H2-K1*) are up-regulated in β-cells and may represent a balance between enhanced antiviral defense and increased immune visibility.

While stimulating responses via enhanced expression of antiviral, antibacterial, and antioxidant genes, there is a dramatic repression of genes that encode proteins involved in maintenance of β-cell identity or those that contribute to β-cell function, such as incretin receptors and ion channels. It has been shown previously that cytokines repress the expression of β-cell identity genes (64, 65), and it has been suggested that this response may confer survival benefits as β-cells that survive in NOD mice have reduced identity gene expression (66). Additionally, β-cell dedifferentiation reduces islet immune cell infiltration and appears to facilitate β-cell escape from immune-mediated destruction (67). Here, we show that these responses occur rapidly in vivo in response to PAMP administration and believe that they contribute to a state of enhanced β-cell fitness by allowing β-cells to evade the adaptive immune system. Importantly, we did not observe expression of dedifferentiation, disallowed, and apoptotic genes 6 h after LPS administration. Taken together, we believe these responses indicate that the early in vivo β-cell response to transient, endogenously produced cytokines is to “fight” and “hide” while preserving the ability to return to a baseline transcriptional profile when cytokine levels return to steady state conditions (31).

In response to PAMP treatment, multiple inflammatory cytokines are produced resulting in elevated serum concentrations (68, 69). To more comprehensively identify genes controlled by IL-1 signaling in islets, we compared the expression of genes up-regulated in β-cells expressing IL-1R following LPS administration to their expression in βIl1r1KO β-cells (Fig 8). Remarkably, of the 1,612 genes differentially expressed (log2FC > 1, *P*-adj < 0.01) following innate immune stimulation with LPS, approximately 1,191 (74%) genes have at least 30% of their expression attributable to IL-1. Functional enrichment analysis of the IL-1 dependent genes reveals that these genes are primarily defense response genes. These results indicate that the changes in β-cell gene expression following innate immune activation are primarily mediated by IL-1, even in the presence of canonical antiviral interferons such as IFN-α, -β, and -γ.

In response to an in vitro treatment of islets with cytokines, the transcriptional responses of α- and δ-cells are similar to those observed in β-cells, suggesting that many cytokine responses are conserved among endocrine cells of the islet (21, 22). Here, we show that this conserved response also exists in vivo, which may be surprising given that the blood supply can differ among islet cell types due to the islet microvasculature and concentrations of cytokines to which the cells are exposed may vary. Greater than 55% of genes up-regulated in α-cells are also up-regulated in β-cells, and many of the remaining genes show increased expression that fell just short of the threshold of significance used for this analysis (log2FC > 1, *P*-adj < 0.01). Given the importance of α- and δ-cells in the regulation of glucose homeostasis, it is logical that these cells would also possess defense mechanisms that can be rapidly initiated by soluble mediators released following recognition of PAMP by the innate immune system. In addition to the conserved gene expression, β-cells up-regulate several genes associated with antiviral defense such as *Gbp5*, *Plscr1*, and *Rsad2* that remain unchanged in α- and δ-cells, indicating some transcriptional responses are unique to the β-cell.

Endothelial cells play a critical role in islet development and function, but their responses to cytokines are relatively unknown. scRNAseq, however, now allows for this type of analysis. Furthermore, we are not aware of any studies examining IEC (IEC) response to endogenously produced cytokines. Knowing that the endothelium is responsive to type 1 interferons, which trigger antiviral responses including increased expression of antipathogen genes, it was not surprising to observe increases in the expression of antipathogen genes in IEC 6 h post LPS administration in mice. However, we did not expect that the primary response of IEC would be a decrease in gene expression. Over three times more IEC genes displayed decreases in expression as compared with the number of genes with an increase in expression in LPS-treated mice. Functional enrichment analysis of genes decreased in expression indicates a broad reduction of transcription by RNA polymerase II and reduced expression of genes involved in key cellular processes such as cell adhesion and migration, lipid metabolic process, angiogenesis, and MAPK signaling. These exciting findings indicate that the response of islets to endogenously produced cytokines is not restricted to endocrine cells but includes the islet endothelium. The observed endothelial cell transcriptional response is likely dependent on not only IL-1, but other soluble mediators such as type 1 interferons. While not a focus of this study, these findings provide unique insight into how IEC may interact with and contribute to endocrine cell survival.

It has been hypothesized that activation of islet resident macrophages and other islet immune cells may contribute to β-cell damage through the production of pro-inflammatory cytokines in islets. In fact, we were the first to report that activated islet macrophages produce and release IL-1 in islets to levels sufficient to stimulate β-cell expression of iNOS and NO-dependent inhibition of insulin secretion (6). Based on our previous studies treating islets in vitro with LPS, one might expect a transcriptional profile of immune cells that is associated with activation including increased levels of the cytokine genes *Il1b*, *Tnf*, and *Il6*. Surprisingly, the expression of these genes appears to be unchanged or modestly decreased in islet immune cells from LPS-treated mice. Instead, we observed increases in the expression of genes encoding proteins that are directly involved in antipathogen defense. These findings suggest these cells are not responding directly to the administered PAMP (as LPS is a potent stimulator of IL-1 and TNF-α expression by macrophages) but are likely responding to soluble mediators known to induce antiviral responses, such as IFNs. We have shown that several interferons, including IFN-α, IFN-β, and IFN-γ, are increased in serum in mice 6-h post LPS administration (31).

IL-1 is not typically thought of as a canonical antiviral cytokine, however, some studies have suggested IL-1 may initiate antiviral responses in certain contexts (70), and we show that cytokine signaling in the β-cell functions to reduce susceptibility to picornavirus infection. Islets from LPS-treated mice have reduced viral mRNA accumulation following infection and treatment of mouse islets with IL-1 or IFN-γ for 6 h decreases EMCV mRNA accumulation by ∼50% in vitro. In combination, the actions of each individual cytokine are additive with greater than 90% reduction in viral mRNA accumulation. Importantly, the antiviral effect of IL-1 requires IL-1 signaling directly in the β-cell as β-cell-specific deletion of IL-1R results in a loss of this antiviral response. These results indicate that pathways beyond interferon signaling, including IL-1-dependendent responses, contribute to β-cell defense against viruses that target β-cells, such as EMCV. Overall, the induction of antipathogen gene expression and the inhibition of picornavirus replication indicate that cytokine signaling in vivo functions to initiate physiological islet responses that reduce the susceptibility of islet cells to pathogens.

## Materials and Methods

### Animals and materials

C57BL/6J mice (RRID:IMSR_JAX:000664) were purchased from The Jackson Laboratory and housed in the MCW Biomedical Resource Center with 14:10 h light/dark cycle. To generate mice with a β-cell specific deletion of Il1r1 (βIl1r1KO), mice expressing Cre in the endogenous Ins1 locus (B6(Cg)-Ins1^tm1.1(cre)Thor^/J; RRID:IMSR_JAX:026801) (71) were bred to mice containing a floxed Il1r1 gene (Il1r1^flox/flox^) with loxP sites positioned to flank exons 3 and 4 (B6.129(Cg)-Il1r1^tm1.1Rbl^/J; The Jackson Laboratory; RRID:IMSR_JAX:028398) (72) as described previously (31). Mice used in this study were males of age 12–16 wk. Animal breeding and experimental procedures were approved by the Institutional Animal Care and Use Committee at the Medical College of Wisconsin.

### Animal treatments and islet isolation

Mice were administered lipopolysaccharide (LPS) from *Escherichia coli* O111:B4 (S-form) (Cat. # IAX-100-012-M001) or endotoxin-free physiological saline (Cat. # IAX-900-003-L010) purchased from Innaxon by IP injection. After 6 h, islets were isolated from mice using methods described previously (73) and immediately dispersed to single cells using 0.1% trypsin (74). Islets from each mouse were processed separately so each replicate represents cells from an individual mouse. Single-cell RNA-sequencing was performed on three biological replicates from C57BL/6J mice (3 saline-treated C57BL/6J mice, 3 LPS-treated C57BL/6J mice) and two biological replicates from βIl1r1KO and littermate control mice (2 saline-treated + 2 LPS-treated βIl1r1KO mice; 2 saline-treated + 2 LPS-treated littermates).

### Single-cell RNA-sequencing

Dispersed islet cells were loaded into Chromium GEM-X Single Cell 3′ Chip, v4 (Cat. # 1000690) and GEMs were generated with the 10X Genomics Chromium Controller. A 3′ gene expression library was constructed using the Chromium GEM-X Single Cell 3′ Kit, v4 (Cat. # 1000691) according to manufacturer instructions. Libraries were sequenced by Novogene Co, Ltd with NovaSeq XPlus 10B PE150 with an average of 44k reads per cell and CellRanger v9 was used to align reads to Mus musculus genome assembly GRCm39. All scRNA-seq analysis was performed using Seurat v5 in R (v4.4.2). The Seurat standard pre-processing workflow was used to filter cells based on the number of unique genes detected in each cell (nFeature_RNA) and cells with greater than 5% mitochondrial genes were excluded as low-quality/dying cells often exhibit high mitochondrial contamination. To capture classical endocrine cell populations while minimizing the occurrence of doublets, cells co-expressing >1 of the following genes were excluded: *Ins2*, *Gcg*, *Sst*, *Ppy*. Note that true bi-hormonal cell populations are rare (75). Additionally, one cluster of low quality cells was excluded from the analysis. The Seurat function sctransform was used to perform normalization, variance stabilization, and feature selection based on a UMI-based gene expression matrix for all samples. DEG tables are provided (Tables S1, S2, S3, S4, S5, S6, S7, and S8) In Fig 8D, the dependence on IL-1 was calculated using the following equation:

$$IL-1\;Dependence=1-\left(\frac{N_{KO.LPS}-N_{KO.Sal}}{N_{Control.LPS}-N_{Control.Sal}}\right)$$

where N_x_ = the normalized count of a given gene from condition x.

Table S1. Differential expression analysis of β-cells following LPS administration to C57BL/6J mice.

Table S2. Differential expression analysis of α-cells following LPS administration to C57BL/6J mice.

Table S3. Differential expression analysis of δ-cells following LPS administration to C57BL/6J mice.

Table S4. Differential expression analysis of endothelial cells following LPS administration to C57BL/6J mice.

Table S5. Differential expression analysis of non-lymphoid hematopoietic cells following LPS administration to C57BL/6J mice.

Table S6. Differential expression analysis of β-cells following LPS administration to Il1r1^flox/flox^ mice.

Table S7. Differential expression analysis of β-cells following LPS administration to βIl1r1KO mice.

Table S8. Differential expression analysis of β-cells following LPS administration to βIl1r1KO versus Il1r1^flox/flox^ mice.

### Islet culture and infection

Islets were cultured at 37^°^C and 5% CO_2_ in Connaught Medical Research Laboratories (CMRL) 1,066 culture media (Thermo Fisher Scientific) containing 5.5 mM glucose and supplemented with 10% heat-inactivated fetal bovine serum (FBS; HyClone), penicillin and streptomycin (Thermo Fisher Scientific), and L-glutamine (Thermo Fisher Scientific). Islets were pre-treated with human recombinant IL-1β (10 U/ml) and murine interferon-γ (150 U/ml) acquired from PeproTech. EMCV-D was obtained as a gift from Dr Ji-Won Yoon (University of Calgary, Calgary, Alberta, Canada). Islets were infected by direct addition to the culture media at a multiplicity of infection (MOI) of five plaque-forming units per cell. Islets were cultured for 1 h, the virus media was replaced with virus-free media, and culture was continued for an additional 11 h.

### RNA isolation and real-time PCR analysis

Total RNA was isolated from islets using Qiagen RNeasy Mini Kit and DNA contamination was removed using TURBO DNA-free Kit (Invitrogen). First strand cDNA synthesis was performed with Maxima H Minus reverse transcriptase (Thermo Fisher Scientific) and oligo(dT)_20_ primers. SsoFast EvaGreen supermix (Bio-Rad) and primers were used to perform quantitative PCR with a Bio-Rad CFX Duet Real-Time PCR System (Bio-Rad). Primers were acquired from Integrated DNA Technologies (Table S9). Gene expression was normalized to Gapdh (ΔC_t_) and expressed as a fold change (2^−ΔΔCt^) relative to control samples.

Table S9. Primer sequences.

### Statistical analysis

*P*-values for differential gene expression were calculated using the Seurat function FindMarkers using the non-parametric Wilcoxon rank sum test. *P*-values were corrected (*P*-adj) for multiple testing with Bonferroni correction. The threshold of significance was set to *P*-adj < 0.01 and log2FC > 1 for all analyses in this study unless otherwise noted. For PCR data, statistical comparisons made between three or more independent conditions were calculated with ANOVA and Tukey’s post hoc analysis. Significant differences are indicated (*P < 0.05).

## Supplementary Material

Reviewer comments

## Data Availability

Sequencing data shown in this publication is available in the National Center for Biotechnology Information (NCBI) Gene Expression Omnibus database under accession number GSE339230.
