# Supplementary material for: Interleukin-1 mediates innate immune signaling and antiviral defense in islets
Source: Life Sci Alliance. 2026 Aug 25;9(11):e202603795. doi: 10.26508/lsa.202603795 (PMC13506774; doi:10.26508/lsa.202603795)
Supplement: Supplementary file 1 [file LSA-2026-03795_TableS9.pdf]

Target	Forward Primer (5'→3')	Reverse Primer (5'→3')
m-GAPDH	GAC ATC AAG AAG GTG GTG AAG C	TCC AGG GTT TCT TAC TCC TTG G
m-EMCV VP1	GGA GTT GAG AAT GCT GAG AG	TTC CAG CAT AAG GAC TTC AG

Supplemental Table 1. qPCR Primers
