# Supplementary material for: Interleukin-1 mediates innate immune signaling and antiviral defense in islets
Source: Life Sci Alliance. 2026 Aug 25;9(11):e202603795. doi: 10.26508/lsa.202603795 (PMC13506774; doi:10.26508/lsa.202603795)
Supplement: Supplementary file 2 [file LSA-2026-03795_review_history.pdf]

# Interleukin-1 mediates innate immune signaling and antiviral defense in islets.

Jacob T. Bartosiak, Polly A. Hansen, Khanmi Kasomva, Sridhar Rao and John A. Corbett
**DOI: https://doi.org/10.26508/lsa.202603795**

*Corresponding author(s): Prof. John A. Corbett (Medical College of Wisconsin)*



## Review Timeline:

| | |
|---|---|
| Submission Date: | 2026-06-02 |
| Editorial Decision: | 2026-07-07 |
| Revision Received: | 2026-07-23 |
| Editorial Decision: | 2026-08-11 |
| Revision Received: | 2026-08-11 |
| Accepted: | 2026-08-17 |

*Scientific Editor: Tim Fessenden*


## Transaction Report:

(Note: With the exception of the correction of typographical or spelling errors that could be a source of ambiguity, letters and reports are not edited. The original formatting of letters and referee reports may not be reflected in this compilation.)

No full Peer Review Process File is available with this article, as the authors have chosen not to make the review process public in this case.

July 7, 2026

Re: Life Science Alliance manuscript #LSA-2026-03795-T

Prof. John A. Corbett
Medical College of Wisconsin
Biochemistry
8701 Watertown plank rd
Milwaukee, Wisconsin 53226

Dear Dr. Corbett,

Thank you for submitting your manuscript entitled "IL-1 is a key in vivo mediator of innate immune signaling and antiviral defense in islets" to Life Science Alliance. The manuscript was assessed by expert reviewers, whose comments are appended to this letter.

As you will see, the reviewers were unanimous in their overall support for this manuscript and the intriguing role for IL-1 signaling identified here. Reviewers 1 and 2 both felt that IL-1-mediated protection against EMCV needed stronger support. We concur this aspect must be strengthened, and we direct you to the suggestion from Reviewer 2 in this regard. Reviewer 2 also noted that potential adverse effects of IL-1 should be discussed, and, if possible, supported with an extended timecourse. Finally, Reviewers 1 and 2 requested methods details and data availability. We invite you to submit a revised manuscript that addresses the above concerns.

I would be happy to discuss the revision in more detail via email or phone/videoconferencing. Please let me know which option you prefer, if any.

To upload the revised version of your manuscript, please log in to your account: https://lsa.msubmit.net/cgi-bin/main.plex

You will be guided to complete the submission of your revised manuscript and to fill in all necessary information. Please get in touch in case you do not know or remember your login name.

The typical timeframe for revisions is three months. Please note that papers are generally considered through only one revision cycle, so strong support from the referees on the revised version is needed for acceptance.

While you are revising your manuscript, please also attend to the below editorial points to help expedite the publication of your manuscript. Please direct any editorial questions to the journal office. When submitting the revision, please include a letter addressing the reviewers' comments point by point.

Thank you for this interesting contribution to Life Science Alliance. We hope that the comments below will prove constructive as your work progresses, and we are looking forward to receiving your revised manuscript.

Sincerely,

Tim Fessenden
Scientific Editor
Life Science Alliance

---------------------------------------------------------------------------

A. THESE ITEMS ARE REQUIRED FOR REVISIONS

-- A letter addressing the reviewers' comments point by point.

-- An editable version of the final text (.DOC or .DOCX) is needed for copyediting (no PDFs).

-- High-resolution figure, supplementary figure and video files uploaded as individual files: See our detailed guidelines for preparing your production-ready images, https://www.life-science-alliance.org/authors

-- Summary blurb (enter in submission system): A short text summarizing in a single sentence the study (max. 200 characters including spaces). This text is used in conjunction with the titles of papers, hence should be informative and complementary to the title and running title. It should describe the context and significance of the findings for a general readership; it should be written in the present tense and refer to the work in the third person. Author names should not be mentioned.

-- By submitting a revision, you attest that you are aware of our payment policies found here: https://www.life-science-alliance.org/copyright-license-fee

B. MANUSCRIPT ORGANIZATION AND FORMATTING:

Full guidelines are available on our Instructions for Authors page, https://www.life-science-alliance.org/authors

We encourage our authors to provide original source data, particularly uncropped/-processed electrophoretic blots and spreadsheets for the main figures of the manuscript. If you would like to add source data, we would welcome one PDF/Excel-file per figure for this information. These files will be linked as supplementary "Source Data" files.

***IMPORTANT: It is Life Science Alliance policy that if requested, original data images must be made available. Failure to provide original images upon request will result in unavoidable delays in publication. Please ensure that you have access to all original microscopy and blot data images before submitting your revision.***

2nd Revision – Editorial Decision August 11, 2026

August 11, 2026

RE: Life Science Alliance Manuscript #LSA-2026-03795-TR

Prof. John A. Corbett
Medical College of Wisconsin
Biochemistry
8701 Watertown plank rd
Milwaukee, Wisconsin 53226

Dear Dr. Corbett,

Thank you for submitting your revised manuscript entitled "Interleukin-1 is a key in vivo mediator of innate immune signaling and antiviral defense in islets." As you can see below, the reviewers have no further requests. We would be happy to publish your paper in Life Science Alliance pending final revisions necessary to meet our formatting guidelines. Thank you for this exciting contribution to the beta-islet field.

MANUSCRIPT ORGANIZATION AND FORMATTING:

To avoid unnecessary delays in the acceptance and publication of your paper, please read the following information carefully. Full guidelines are available on our Instructions for Authors page, https://www.life-science-alliance.org/authors

**Submission of a paper that does not conform to Life Science Alliance guidelines will delay the acceptance of your manuscript.**

-While the current title is acceptable, we suggest the following changes for brevity and clarity: "Interleukin-1 mediates innate immune signaling and antiviral defense in pancreatic islets"
-Please add the X and Bluesky handles of your host institute/organization, as well as your own, and/or one of the authors, in our system.
-The contributions selected for Sridhar Rao do not qualify them for authorship. Please either update the contributions in our system and in the Author Contributions section of the manuscript, or let us know if the author should be removed (and added potentially to the acknowledgment section).
-Please add your main, supplementary figure, and table legends to the main manuscript text after the references section.
-Please add callouts for Figures S1A-G; S5A-F and S6A-G to your main manuscript text.
-We suggest you upload Figure 11 as a Graphical Abstract instead of a figure file, so that it will appear online adjacent to the abstract. This is however left to your discretion.

We encourage our authors to provide original source data, particularly uncropped/-processed electrophoretic blots and spreadsheets for the main figures of the manuscript. If you would like to add source data, we would welcome one PDF/Excel-file per figure for this information. These files will be linked as supplementary "Source Data" files.

It is Life Science Alliance policy that if requested, original data images must be made available to the editors. Failure to provide original images upon request will result in unavoidable delays in publication. Please ensure that you have access to all original data images prior to final submission.

If you are planning a press release on your work, please inform us immediately to allow informing our production team and scheduling a release date.

We welcome submissions of potential cover images for the issue of LSA in which your work would appear. If you have high quality images associated with this work, please feel free to email these, with a caption, to the journal office.

LSA encourages authors to provide a 30-60 second video where the study is briefly explained. These videos will be appear embedded with the manuscript online at Life Science Alliance, and on social media to promote the published paper and authors (for examples, see https://docs.google.com/document/d/1-UWCfbE4pGcDdcgzcmiuJl2XMBJnxKYeqRvLLrLSo8s/edit?usp=sharing). Corresponding or first-authors are welcome to submit the video. Please submit only one video per manuscript. The video can be emailed to contact@life-science-alliance.org

FINAL FILES:

To upload the final version of your manuscript, please log in to your account: https://lsa.msubmit.net/cgi-bin/main.plex
You will be guided to complete the submission of your revised manuscript and to fill in all necessary information. Please get in touch in case you do not know or remember your login name.

The following items are required for acceptance.

-- An editable version of the final text (.DOC or .DOCX) is needed for copyediting (no PDFs).

-- High-resolution figure, supplementary figure and video files uploaded as individual files: See our detailed guidelines for preparing your production-ready images, https://www.life-science-alliance.org/authors

-- Summary blurb (enter in submission system): A short text summarizing in a single sentence the study (max. 200 characters including spaces). This text is used in conjunction with the titles of papers, hence should be informative and complementary to the title. It should describe the context and significance of the findings for a general readership; it should be written in the present tense and refer to the work in the third person. Author names should not be mentioned.

**The license to publish form must be signed before your manuscript can be sent to production. A link to the license to publish form will be available to the corresponding author only. Please take a moment to check your funder requirements.**

**Reviews, decision letters, and point-by-point responses associated with peer-review at Life Science Alliance will be published, alongside the manuscript. If you do want to opt out of having the reviewer reports and your point-by-point responses displayed, please let us know immediately.**

Thank you for your attention to these final processing requirements. Please revise and format the

manuscript and upload materials as soon as you are able.

Thank you for this interesting contribution to the literature. We look forward to publishing your paper in Life Science Alliance.

Sincerely,

Tim Fessenden
Scientific Editor
Life Science Alliance

August 17, 2026

RE: Life Science Alliance Manuscript #LSA-2026-03795-TRR

Prof. John A. Corbett
Medical College of Wisconsin
Biochemistry
8701 Watertown plank rd
Milwaukee, Wisconsin 53226

Dear Dr. Corbett,

Thank you for submitting your Research Article entitled "Interleukin-1 mediates innate immune signaling and antiviral defense in islets.". It is a pleasure to let you know that your manuscript is now accepted for publication in Life Science Alliance. Congratulations on this interesting work!

The final published version of your manuscript will be deposited by us to PubMed Central upon publication.

Your manuscript will now progress through copyediting and proofing. It is journal policy that authors provide original data upon request.

Your article will publish open access upon publication under a CC-BY license.

Reviews, decision letters, and point-by-point responses associated with peer-review at Life Science Alliance will be published, alongside the manuscript. If you do want to opt out of having the reviewer reports and your point-by-point responses displayed, please let us know immediately.

***IMPORTANT: If you will be unreachable at any time, please provide us with the email address of an alternate author. Failure to respond to routine queries may lead to unavoidable delays in publication.***

Scheduling details will be available from our production department. You will receive proofs shortly before the publication date. Only essential corrections can be made at the proof stage so if there are any minor final changes you wish to make to the manuscript, please let the journal office know now.

DISTRIBUTION OF MATERIALS:
Authors are required to distribute freely any materials used in experiments published in Life Science Alliance. Authors are encouraged to deposit materials used in their studies to the appropriate repositories for distribution to researchers.

You can contact the journal office with any questions, contact@life-science-alliance.org

Again, congratulations on a very nice paper. I hope you found the review process to be constructive and are pleased with how the manuscript was handled editorially. We look forward to future exciting submissions from your lab.

Sincerely,

Tim Fessenden
Scientific Editor
Life Science Alliance
